# Epithelial Cell Rests of Malassez Provide a Favorable Microenvironment for Ameliorating the Impaired Osteogenic Potential of Human Periodontal Ligament Stem Cells

**DOI:** 10.3389/fphys.2021.735234

**Published:** 2021-10-11

**Authors:** Yanjiao Li, Anqi Liu, Liqiang Zhang, Zhiwei Wang, Nana Hui, Qiming Zhai, Lishu Zhang, Zuolin Jin, Fang Jin

**Affiliations:** ^1^State Key Laboratory of Military Stomatology & National Clinical Research Center for Oral Diseases & Shaanxi Clinical Research Center for Oral Diseases, Department of Orthodontic, School of Stomatology, The Fourth Military Medical University, Xi'an, China; ^2^Department of Stomatology, The 985 Hospital of PLA, Taiyuan, China; ^3^Xi'an Institute of Tissue Engineering and Regenerative Medicine, Xi'an, China; ^4^State Key Laboratory of Military Stomatology & National Clinical Research Center for Oral Diseases & Shaanxi International Joint Research Center for Oral Diseases, Center for Tissue Engineering, School of Stomatology, The Fourth Military Medical University, Xi'an, China

**Keywords:** periodontal ligament stromal/stem cells, epithelial cell rests of Malassez, osteogenesis, co-culture, Wnt pathway

## Abstract

Human periodontal ligament stromal/stem cells (PDLSCs) are ideal candidates for periodontal regeneration and are of significant importance in clinical practice. However, PDLSCs derived from diseased microenvironments exert impaired behavior, which leads to the failure of periodontal regeneration. The epithelial cell rests of Malassez (ERM), which are involved in periodontal homeostasis, are residual cells from Hertwig's epithelial root sheath (HERS). However, the function of ERM remains largely unknown. Therefore, the aim of this study was to evaluate the effect of ERM on the osteogenic potential of PDLSCs from an impaired microenvironment. PDLSCs from healthy donors (H-PDLSCs), periodontitis donors (P-PDLSCs) and human ERM were harvested. Osteogenic evaluation showed a lower osteogenic potential of P-PDLSCs compared to that of H-PDLSCs. Then, we co-cultured ERM with P-PDLSCs, and the data showed that ERM promoted the expression of osteogenic genes and proteins in P-PDLSCs. In addition, we collected the PDLSCs from aged donors (A-PDLSCs) and analyzed the osteogenesis capacity of the A-PDLSCs and A-PDLSCs + ERM groups, which displayed similar results to P-PDLSCs. Finally, we evaluated the Wnt pathway, which is associated with osteogenic differentiation of stromal/stem cells, in A-PDLSCs + ERM and P-PDLSCs + ERM groups, which indicated that suppression of the Wnt pathway may result in an increase in the osteogenic properties of A-PDLSCs + ERM and P-PDLSCs + ERM groups. Taken together, the above findings shed new light on the function of ERM and provide a novel therapeutic for optimizing PDLSCs-based periodontal regeneration.

## Introduction

Periodontal regeneration has always been widely investigated in periodontal field (Liu et al., 2018; Behm et al., 2020; Wang et al., 2020). Due to the complex periodontal ligament structure and oral environment, satisfactory periodontal regeneration still has not achieved (Li et al., 2020; Nibali et al., 2021; Shang et al., 2021a). Human periodontal ligament stromal/stem cells (PDLSCs), which are a kind of mesenchymal stromal/stem cells (MSCs) have been widely used in periodontal tissue regeneration (Liu et al., 2018). However, studies have recognized that purely applying PDLSCs barely regenerates ideal periodontal tissue. Therefore, numerous studies have focused on facilitating the capacities of PDLSCs to improve the effect of periodontal regeneration (Shang et al., 2017, 2021b; Abdelaziz et al., 2021).

The periodontal ligament is a complex tissue that maintains various cell types such as PDLSCs, fibroblasts and epithelial cell rests of Malassez (ERM) (LeBlanc et al., 2020). Among them, ERM are the remaining epithelial cells of Hertwig's epithelial root sheath (HERS), which play important roles in maintaining the homeostasis of periodontal tissue (Yang et al., 2015) and preventing root resorption (Tsunematsu et al., 2016). However, it remains unclear how ERM impacts PDLSCs. The microenvironment is involved in regulating the biological behavior of stromal/stem cells (Sui et al., 2019). For instance, the osteogenic capacity of PDLSCs derived from the inflammatory microenvironment is lower than that of PDLSCs from a healthy microenvironment (Tang et al., 2016). Thus, the alleviation of the impaired microenvironment would be helpful for enhancing the results of periodontal regeneration.

In this study, we established a co-culture system to observe the effect of ERM on the osteogenesis of PDLSCs derived from an impaired microenvironment., thereby providing a theoretical basis for further optimization of periodontal regeneration.

## Materials and Methods

### Cell Isolation and Culture

Healthy PDLSCs (H-PDLSCs) were obtained by culturing explants of healthy periodontal tissues from patients (24–35 years of age) whose premolar or third molar was extracted for orthodontic reasons. Periodontitis PDLSCs (P-PDLSCs) were obtained from patients (24–35 years of age) diagnosed with periodontitis who exhibited 2/3 alveolar bone loss and more than one pocket with a depth ≥5 mm. Aged PDLSCs (A-PDLSCs) were collected from patients aged 52–65 years. Periodontal tissues were gently separated from the root surface. After washing in sterile phosphate-buffered solution (PBS), the tissues were digested with type I collagenase (0.66 mg/mL; Sigma, USA) for 20 min. Single cell suspensions were generated and cultured in culture medium containing α-minimum essential medium (α-MEM; Gibco, USA) supplemented with 10% fetal bovine serum (FBS; Thermo Electron, USA), 0.292 mg/mL glutamine (*Invitrogen*, USA), 100 U/mL penicillin, 100 mg/mL streptomycin (Gibco, USA) at 37°C in a humidified atmosphere of 5% CO_2_ and 95% air.

To obtain human ERM, 15 systemically healthy donors aged between 11 and 30 years were recruited. Healthy premolars or third molars were extracted for orthodontic reasons. The first third of the tooth which proximately to the crown was removed. After thoroughly scraping the periodontal tissue of upper 1/3 of root to remove the gingival tissue, the tooth was washed in sterile PBS and totally digested with type I collagenase (0.66 mg/mL; Sigma, USA) for 2 h at 37°C in a humidified atmosphere of 5% CO_2_ and 95% air. Single cell suspensions were generated by filtration using a 70 μm strainer, then washed and re-suspended in α-MEM (Gibco, USA) supplemented with 15% FBS (Thermo Electron, USA), 0.292 mg/mL glutamine (*Invitrogen*, USA), 100 U/mL penicillin, and 100 mg/mL streptomycin (Gibco, USA). The culture medium was changed every 3 days. The explants were maintained in 6-well culture dishes for 2 weeks until cell clusters were formed. After digestion, cells were seeded and cultured with epithelial cell medium consisting of α-MEM (Gibco, USA), 2% FBS (Thermo Electron, USA), 1% epithelial cell growth supplement (Gibco, USA), and 1% penicillin/streptomycin (Gibco, USA) for 7 days. Then, differential digestion was performed using trypsin-EDTA (9:1) for 10 min to detach the fibroblast-like-cells.

All samples were collected at the School of Stomatology, The Fourth Military Medical University. Written informed consent was provided by all participants, and the study was approved by the hospital's ethics committee.

### Osteogenic Differentiation Assay

The induction of osteogenic differentiation was performed as previously described (Li et al., 2016). Briefly, PDLSCs in passage 3–4 were used for osteogenic differentiation assay. 2 × 10^5^ cells per well were maintained in 6-well plates with culture medium. When the cells reached ~90% confluence, the culture medium was changed to osteoinductive medium which supplemented with 100 nM dexamethasone, 50 mg/ml ascorbic acid, and 5 mM b-glycerophosphate (Sigma, USA).

After 7 days of osteogenic induction, cells were fixed in 4% paraformaldehyde for 30 min. Subsequently, alkaline phosphatase (ALP) staining was performed using the BCIP/NBT Alkaline Phosphatase Kit (Beyotime, China). In addition, total RNA and protein were extracted and analyzed for the expression of osteogenic genes (ALP and RUNX2) and proteins (ALP and RUNX2).

### Immunofluorescence Staining

ERM were fixed in 4% paraformaldehyde and incubated in 0.1% Triton X-100 for 15 min. Next, the cells were incubated overnight with a rabbit anti-human CK14 primary antibody (Abcam, 1:200) at 4°C and then the secondary antibody (Jackson, 1:200) was applied to react with the primary antibody. The cell nuclei were stained with DAPI (Sigma, 1:200) and observed under fluorescence microscope.

### Co-culture System

The coculture system was established as previously described (Liu et al., 2014a). Cell coculture assay was assessed using 12-well inserts with 0.4-μm pores (Corning, China) according to the manufacturer's protocol. Briefly, ERM were loaded into the upper chamber (5.5 × 10^3^), while P-PDLSCs or A-PDLSCs were loaded into the lower chamber (4.0 × 10^4^) in osteoinductive medium.

### Quantitative Real-Time PCR (qRT-PCR)

To compare gene expression, qRT-PCR was used according to protocol. After 7 days of osteogenic induction, cells were washed with PBS and total RNA was extracted. Synthesis of cDNA was performed using SYBR1 Premix Ex Taq II (Perfect Real Time kit; TaKaRa, Japan). The primers are listed below:

*GAPDH:* Forward *5*′*- TCT GCA TCA TCC AGG AGC TTA TT*−*3*′, Reverse *5*′*- TGA TAC AGA AGG CAG GTT CAC AA*−*3*′*; ALP:* Forward *5*′*- AGC TTT CGA AGA ACA ACG GA*−*3*′, Reverse *5*′*- TCT TGA AAT GCT TTG GGT CC-3*′*; RUNX2:* Forward *5*′*- CCC GTG GCC TTC AAG GT*−*3*′, Reverse *5*′*- CGT TAC CCG CCA AGA CAG TA-3*′.

### Western Blot Analysis

After 7 days of osteogenic induction, cells were washed with PBS and total protein was obtained by RIPA lysis buffer (Beyotime, China). Quantitative analysis was performed using BCA (Sigma, USA). Next, proteins were isolated on NuPAGE 10–12% polyacrylamide gel and transferred to a PVDF membrane (Millipore, USA). The membrane was blocked with 5% milk for 2 h and then incubated with primary antibodies overnight. The following primary antibodies were used: ALP (Abcam, 1:400), RUNX2 (Abcam, 1:400), total-β-catenin (Abcam, 1:1000), active-β-catenin (Cell Signaling, 1:500), GSK-3β (Abcam, 1:1000), p-GSK-3β (Santa Cruz, 1:500) and GAPDH (Cwbiotech, 1:500). The secondary antibody (Corning, 1:5000) was incubated according to the source of primary antibody, and the chemiluminescence ECL kit (Sigma, USA) was used for protein detection. ImageJ was used to analyze the corresponding spectral band intensity of scanned images.

### Statistical Analysis

Statistical analysis was performed using GraphPad Prism 8.0. The results are expressed as the mean ± SD from at least three independent experiments and analyzed by two-tailed unpaired Student's *t*-test. A value of *P* < 0.05 was considered statistically significant.

## Results

### PDLSCs From the Inflammatory Microenvironment Display Impaired Osteogenic Potential

First, to identify the MSCs properties of PDLSCs, we analyzed the surface markers of PDLSCs from healthy (H-PDLSCs) and inflammatory (P-PDLSCs) microenvironments. Flow cytometry evaluation showed that H-PDLSCs and P-PDLSCs were both positive for the MSCs surface markers CD105, CD90 and CD29, but were negative for the hematopoietic marker CD45 (Supplementary Figure S1A). In addition, they both possessed colony-formation capacity (Supplementary Figure S1B), positive for ALP activity staining (Supplementary Figure S1C) and formed osteogenic nodules, as shown through Alizarin red S staining (Supplementary Figure S1D).

**Figure 1:**
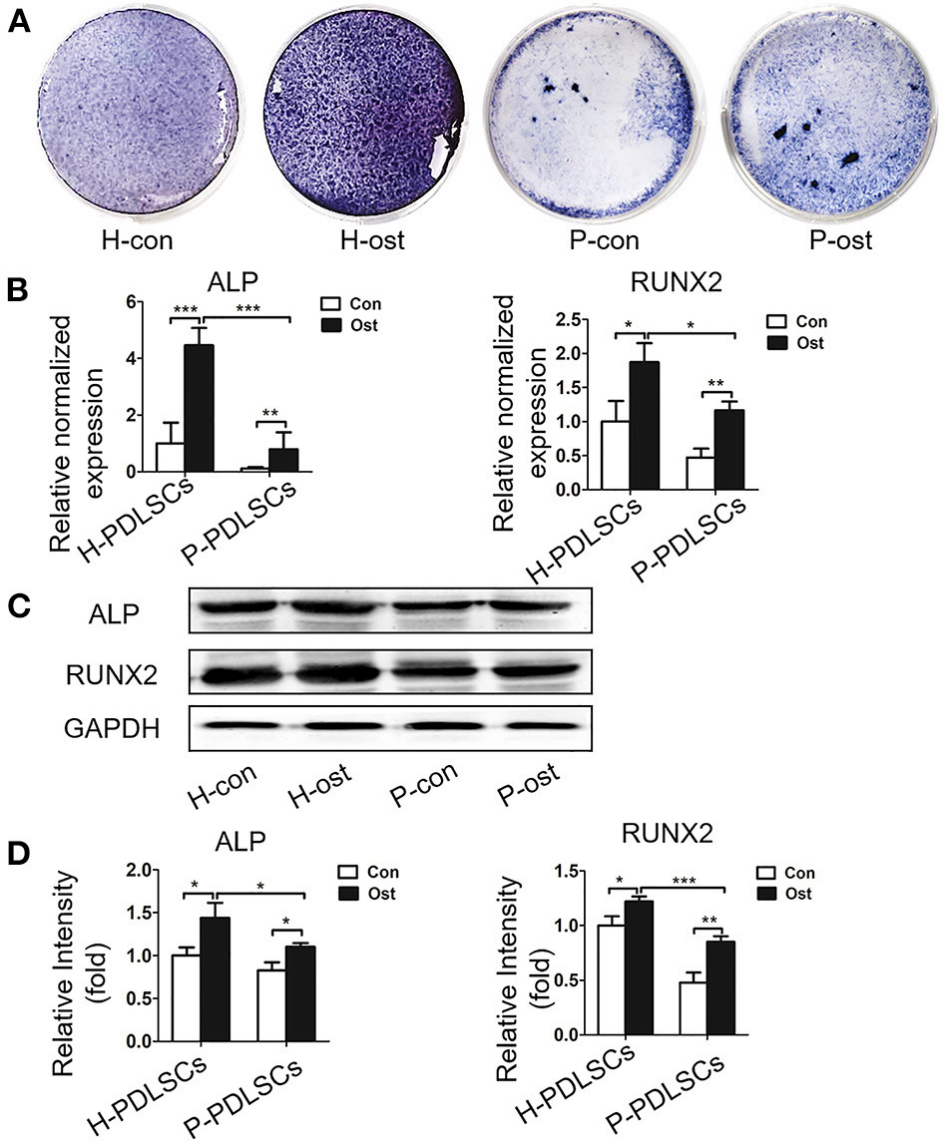
PDLSCs from the inflammatory microenvironment display impaired osteogenic potential. **(A)** ALP staining shows that ALP positive staining reduced in P-PDLSCs, comparing to H-PDLSCs, after 7 days of osteogenic induction (*n* = 3). **(B)** The qRT-PCR results display higher expression levels of the osteogenic genes ALP and RUNX2 after osteogenic induction in both P-PDLSCs and H-PDLSCs than in PDLSCs without osteogenic induction, while the expression of ALP and RUNX2 decrease in P-PDLSCs after 7 days of osteogenic induction (*n* = 3). **(C)** Expression of ALP and RUNX2 are examined by Western blot analysis. **(D)** The quantitative evaluation of Western blot demonstrates that compare to H-PDLSCs, the expression levels of ALP and RUNX2 reduce in P-PDLSCs (*n* = 3). *P < 0.05, **P < 0.01, ***P < 0.001.

Next, the osteogenic capacity of H-PDLSCs and P-PDLSCs was compared after 7 days of osteogenic induction. There were four groups: H-PDSLCs with (H-ost) or without osteogenic induction (H-con) and P-PDSLCs with (P-ost) or without osteogenic induction (P-con). ALP staining showed that the expression level of ALP increased after osteogenic induction in both H-PDLSCs and P-PDLSCs. The ALP expression level was obviously decreased in the P-ost group compared with the H-ost group (Figure 1A). Additionally, mRNA expression of the osteogenic genes ALP and RUNX2, which are classical markers of osteogenesis, was determined by qRT-PCR, and their protein expression levels were assessed by Western blot. The results showed that osteogenic induction enhanced the ALP and RUNX2 gene expression of PDLSCs and that the P-PDLSCs exhibited lower gene expression levels than H-PDLSCs after osteogenic induction (*P* < 0.001; Figure 1B), which is consistent with the Western blot results (^*^P < 0.05, ^***^P < 0.001; Figures 1C,D). The findings revealed that the P-PDLSCs showed impaired osteogenic ability, which was consistent with previous results (Sun et al., 2017), thereby indicating that the inflammatory microenvironment impairs the osteogenic capacity of PDLSCs.

### ERM Promote the Osteogenic Potential of PDLSCs From Inflammatory Microenvironment

**Figure 2:**
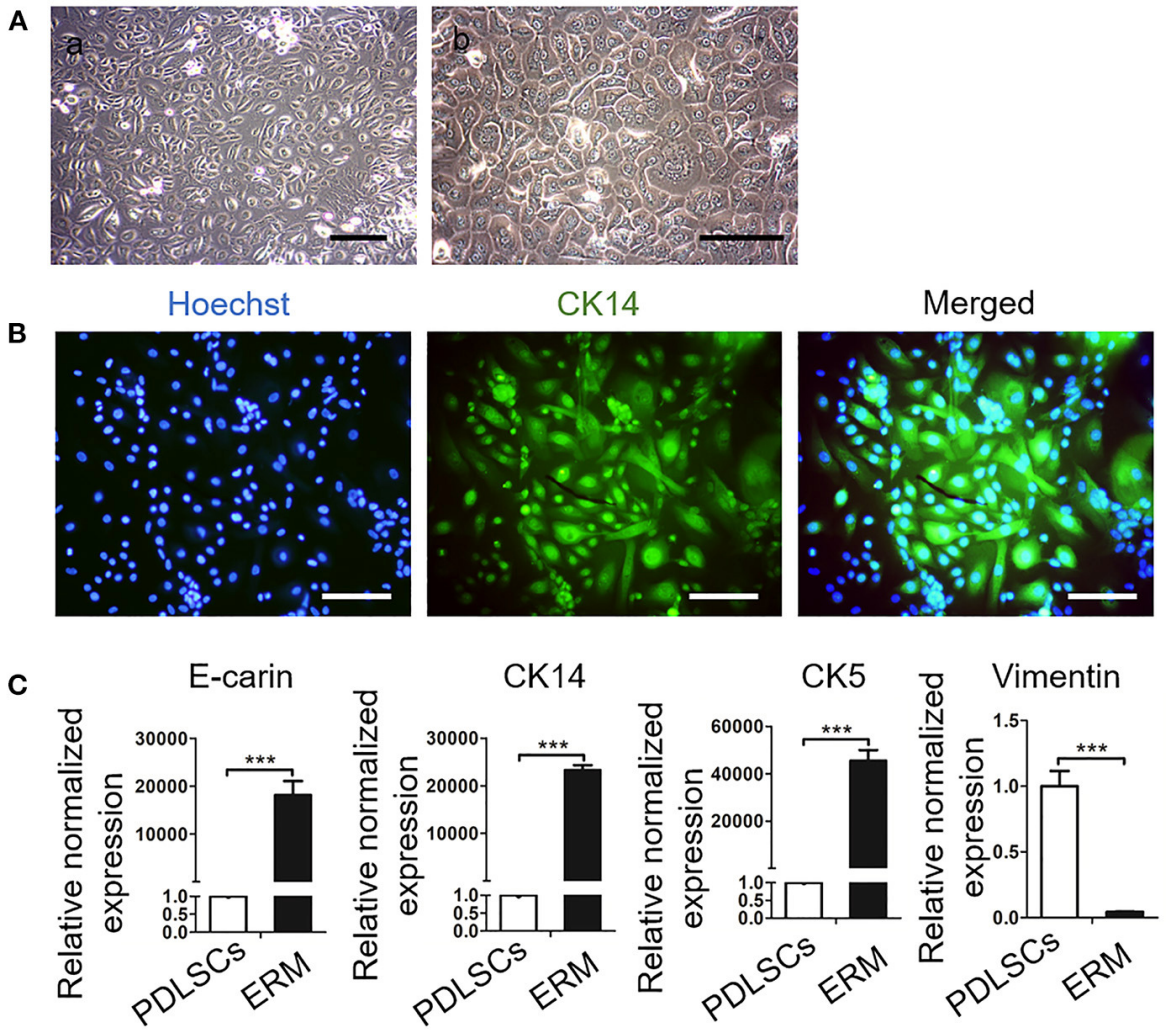
Culture and Identification of epithelial rests of Malassez (ERM). **(A)** Representative morphologies of ERM. (a) Low magnification of ERM. Scale bar, 200 mm; (b) High magnification of ERM; Scale bar, 20 mm. **(B)** Immunofluorescence staining shows that the cells are positive for the epithelial specific marker CK14. Scale bar, 50 mm. **(C)** qRT-PCR reveals that ERM cells express epithelial specific markers CK14, CK5 and E-cadrin, but barely express PDLSCs specific marker vimentin (*n* = 3). ***P < 0.001.

It has been reported that ERM express extracellular matrix proteins that regulate the function of PDLSCs and maintain PDL homeostasis (Keinan and Cohen, 2013; Xiong et al., 2013). In this study, ERM were successfully isolated and purified from periodontal tissues and displayed a “paving stone” shape (Figure 2A). Immunofluorescence showed ERM were positive for the epithelial marker cytokeratin 14 (CK14) (Figure 2B). qRT-PCR showed high expression levels of the epithelial markers amelogenin, CK14, cytokeratin 5 E-cadherin in ERM, but these were poorly detected in PDLSCs. Additionally, ERM barely expressed in MSCs marker vimentin which was highly expressed in PDLSCs (*P* < 0.001; Figure 2C). The findings showed that the ERM we cultured possessed the characteristics of epithelial cells which can be applied for further studies.

**Figure 3:**
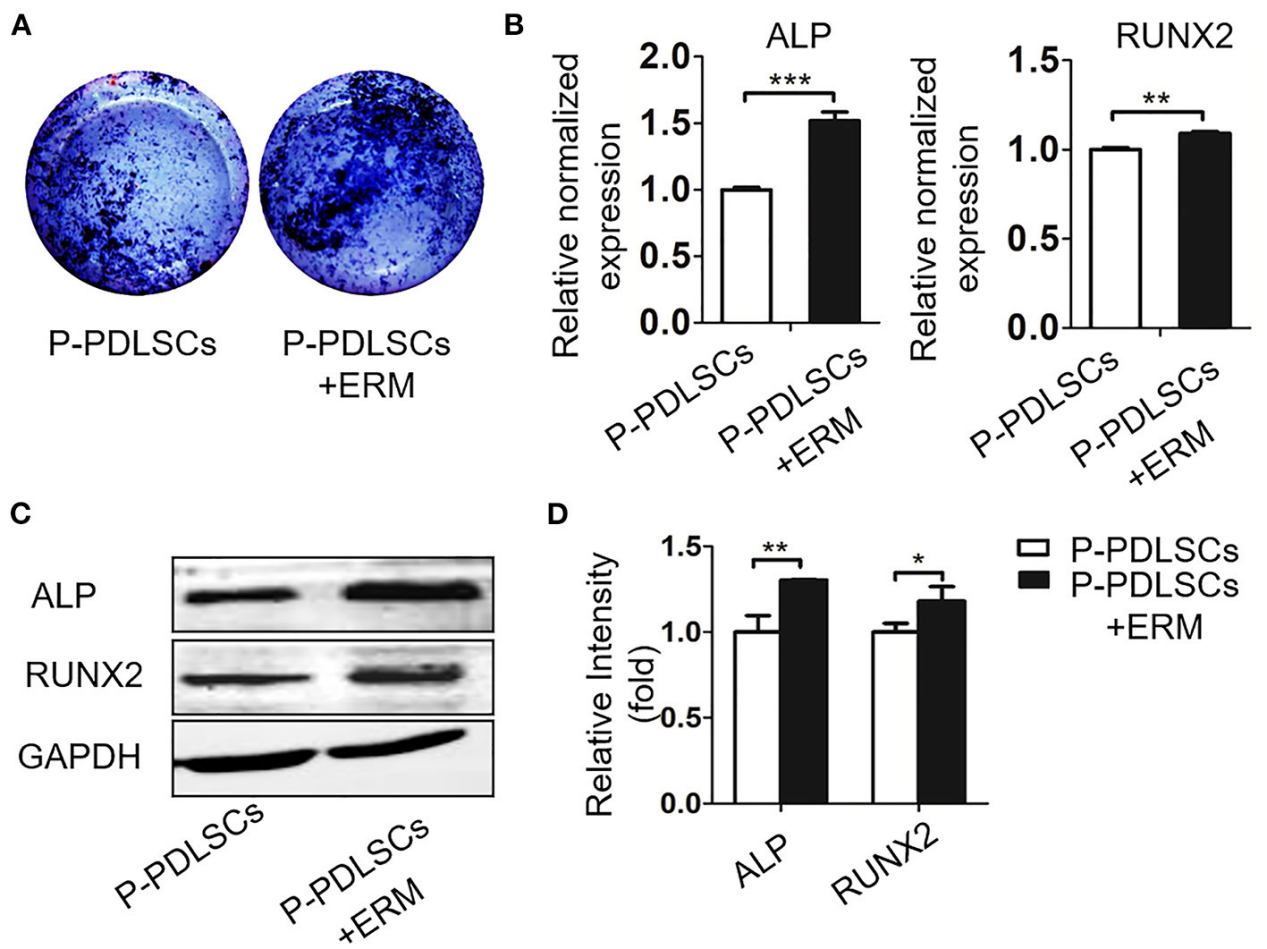
ERM promote the osteogenic potential of PDLSCs from inflammatory microenvironment. **(A)** ALP staining exhibits that co-cultured with ERM elevates the ALP expression of P-PDLSCs after osteogenic induction. **(B)** qRT-PCR analysis shows P-PDLSCs + ERM group expresses higher levels of osteogenic genes ALP and RUNX2 than P-PDLSCs group (*n* = 3). **(C)** Protein expression levels of ALP and RUNX2 also increase in P-PDLSCs + ERM group. **(D)** The quantitative evaluation of Western blot (*n* = 3). *P < 0.05, **P < 0.01, ***P < 0.001.

To assess the effect of ERM on P-PDLSCs, we established an osteogenic co-culture system. ALP staining showed that higher ALP expression in the P-PDLSCs and ERM co-culture group (P-PDLSCs + ERM) than P-PDLSCs group (Figure 3A). The mRNA expression of ALP and RUNX2, which are associated with osteogenesis, increased in P-PDLSCs + ERM group (^**^P < 0.01, ^***^P < 0.001; Figure 3B). However, the fold change of RUNX2 is lower than that of ALP after statistical analysis which we will confirm in future studies. The Western blot findings also displayed increased expression levels of ALP and RUNX2 in the P-PDLSCs + ERM group, consisting with qRT-PCR findings (^*^P < 0.05, ^**^P < 0.01; Figures 3C,D). The data above first determined that ERM co-cultured with P-PDLSCs enhanced the osteogenic capacity of P-PDLSCs, implying that ERM may create a favorable microenvironment to rescue the impaired osteogenic properties of P-PDLSCs.

### ERM Enhance the Osteogenic Ability of PDLSCs From Aged Microenvironment

**Figure 4:**
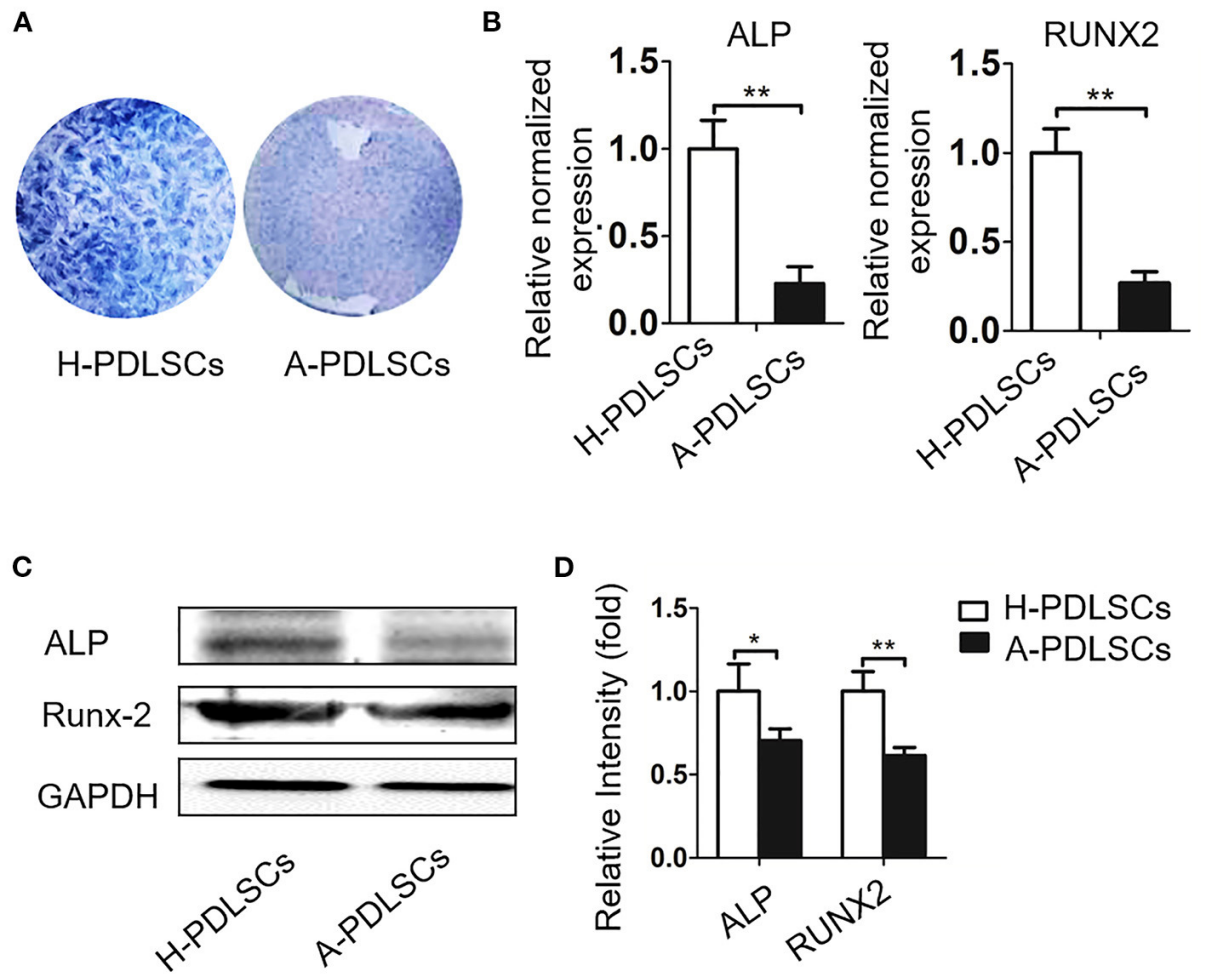
The PDLSCs from aged microenvironment exhibit impaired osteogenic capacity. **(A)** ALP staining shows that after osteogenic induction, ALP positive staining reduced in A-PDLSCs, comparing to H-PDLSCs (*n* = 3). **(B)** qRT-PCR demonstrates lower expression of osteogenic genes ALP and RUNX2 in A-PDLSCs than H-PDLSCs (*n* = 3). **(C)** Expression of the ALP and RUNX2 are examined by Western blot analysis. **(D)** The quantitative evaluation of Western blot shows that P-PDLSCs express lower level of ALP and RUNX2 than H-PDLSCs (*n* = 3). *P < 0.05, **P < 0.01.

Given that cells from unfavorable microenvironment exhibited impaired biological characteristics (Sui et al., 2019), we further analyzed osteogenic capacity of PDLSCs from aged microenvironment (A-PDLSCs). Flow cytometry analysis showed that A-PDLSCs were positive for the MSCs surface markers CD105, CD90 and CD29, but were negative for the hematopoietic marker CD45 (Supplementary Figure S2A). Besides, A-PDLSCs possessed colony-formation (Supplementary Figure S2B), ALP positive staining (Supplementary Figure S2C), and osteogenic nodule formation potential (Supplementary Figure S2D). After seven days of osteogenic induction, the ALP staining showed the reduced expression of ALP in A-PDLSCs compared to H-PDLSCs (Figure 4A). The gene and protein expression of ALP and RUNX2, which are classical markers of osteogenesis, were both decreased in A-PDLSCs (^*^P < *0.05*, ^**^P < *0.01;* Figures 4B-D). The data determined that PDLSCs from aged microenvironment exhibit impaired osteogenic potential, consistent with previous findings.

**Figure 5:**
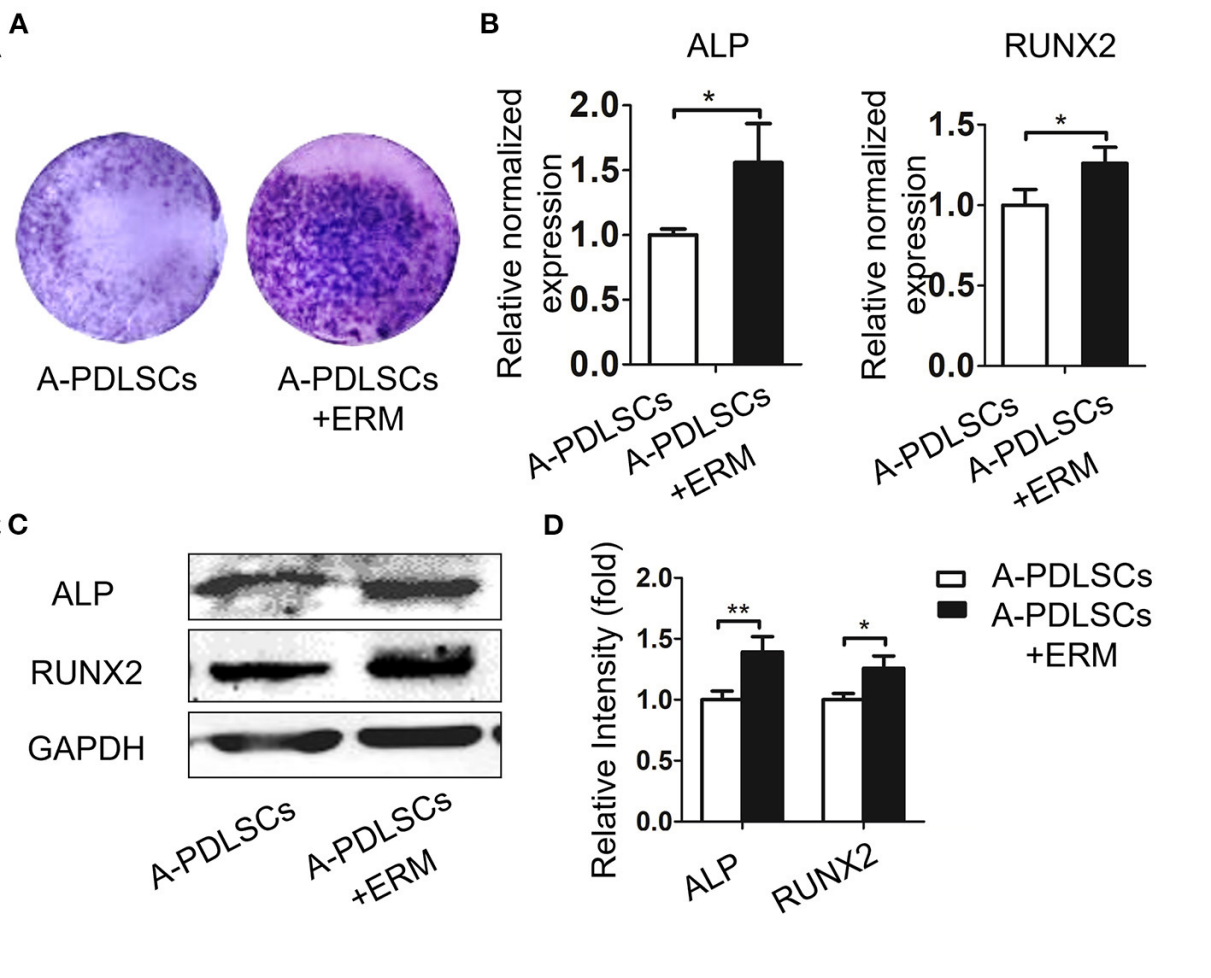
ERM enhance the osteogenic ability of PDLSCs from aged microenvironment. **(A)** ALP staining exhibits that co-cultured with ERM elevates the ALP expression of A-PDLSCs after osteogenic induction. **(B)** qRT-PCR analysis shows that the A-PDLSCs + ERM group expresses higher levels of ALP and RUNX2 than the A-PDLSCs group (*n* = 3). **(C)** Protein expression levels of ALP and RUNX2 also increase in the A-PDLSCs + ERM group. **(D)** The quantitative evaluation of Western blot (*n* = 3). *P < 0.05, **P < 0.01.

Since ERM co-cultured with P-PDLSCs alleviated the reduced osteogenic property of P-PDLSCs, we next analyzed the osteogenesis capacity of A-PDLSCs + ERM. ALP staining displayed an increase in ALP expression in the A-PDLSCs + ERM group after 7 days of osteogenic induction (Figure 5A). Comparing to A-PDLSCs group, the gene and protein expression levels of ALP and RUNX2 also increased in A-PDLSCs + ERM group (^*^P < *0.05*, ^**^P < *0.01;* Figures 5B-D). The results first showed that ERM promote the osteogenic ability of PDLSCs from aged microenvironment, indicating that ERM may provide a beneficial microenvironment for PDLSCs to elevate their impaired properties.

### ERM Alleviate the Osteogenic Property of PDLSCs From Impaired Microenvironment via Suppressing the Wnt Pathway

**Figure 6:**
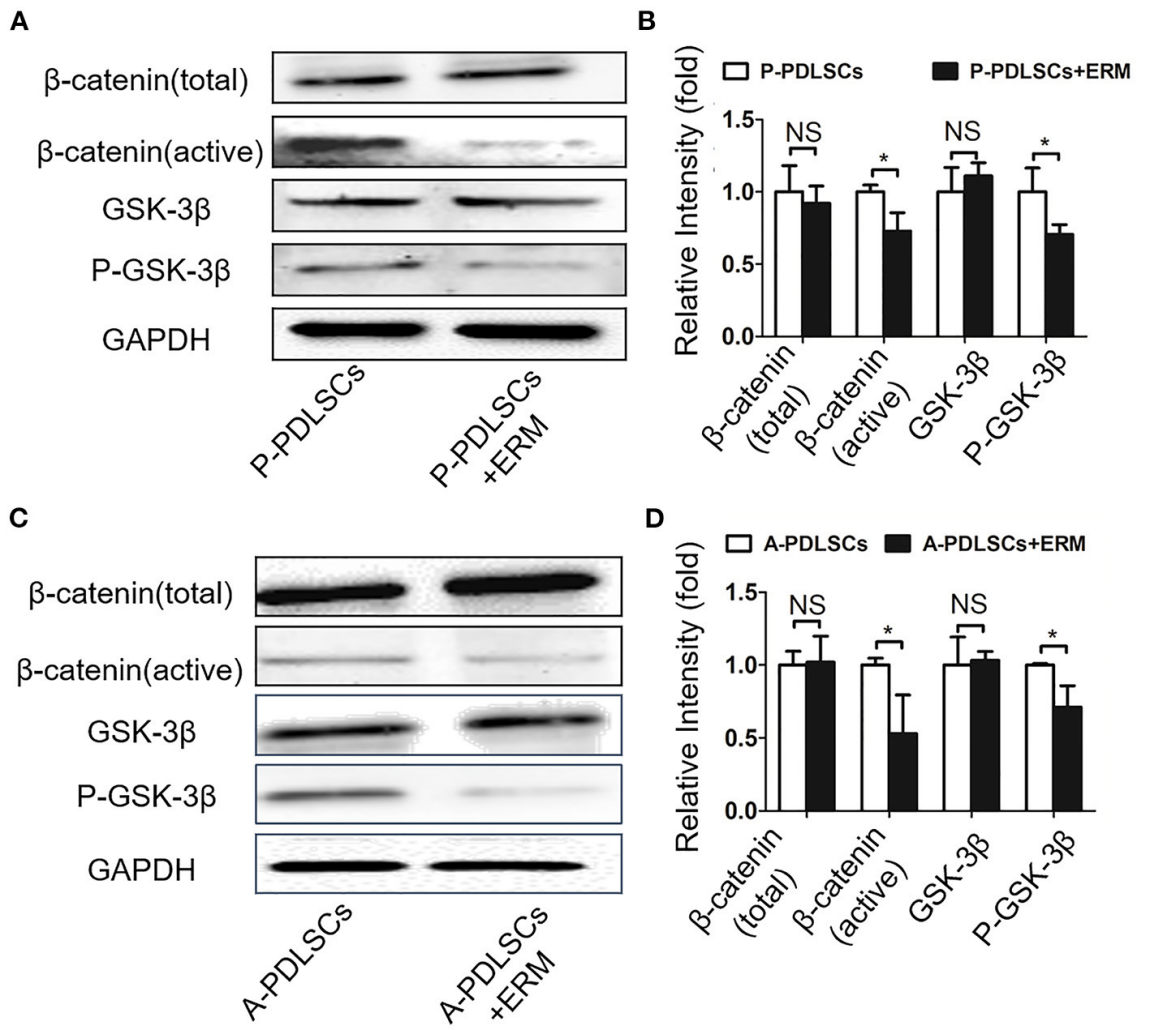
ERM alleviate the osteogenic property of PDLSCs from impaired microenvironment via suppressing the Wnt pathway. **(A)** Western blot analysis exhibits that the expression levels of active-β-catenin and P-GSK-3β decrease in P-PDLSCs + ERM group. **(B)** Quantitative evaluation of Western blot (*n* = 3). **(C)** Western blot analysis shows that the expression levels of active-β-catenin and P-GSK-3β also decrease in the A-PDLSCs + ERM group. **(D)** Quantitative evaluation of Western blot (*n* = 3). *P < 0.05.

Recent studies have demonstrated that the Wnt signaling pathway plays an important role in the osteogenic differentiation of PDLSCs (Liu et al., 2011a). However, it is still elusive whether ERM alleviate the impaired osteogenic ability of PDLSCs which were from unfavorable microenvironment via Wnt signaling. In this study, we evaluated β-catenin and GSK-3β, which are key proteins in the Wnt pathway. The findings showed that the GSK-3β and total-β-catenin expression levels were essentially unchanged, while P-GSK-3β and active-β-catenin protein expression levels in the P-PDLSCs + ERM group were lower than those in the P-PDLSCs group (*P* < *0.05*; Figures 6A,B). Furthermore, the Wnt pathway in A-PDLSCs + ERM and A-PDLSCs group was also analyzed. The results displayed that there was no significant difference in GSK-3β and total-β-catenin expression levels, but that P-GSK-3β and active-β-catenin protein expression levels decreased in the A-PDLSCs + ERM group (*P* < *0.05*; Figures 6C,D). The results revealed that the Wnt pathway was inhibited in PDLSCs from impaired microenvironment when they were co-cultured with ERM, which may be associated with their enhanced osteogenic properties. However, further studies are needed to confirm the findings.

## Discussion

A favorable microenvironment promotes the biological properties of PDLSCs, which leads to better outcomes of periodontal regeneration (Liu et al., 2018; Zhou et al., 2021). Our results first found that ERM enhance the osteogenic capacity of PDLSCs from inflammatory and aging microenvironments, providing a novel role of ERM and a new thought for optimizing periodontal regeneration.

The extracellular microenvironment tightly regulates the biological behavior of resident stem cells (Sui et al., 2019). For instance, the efficacy of periodontal regeneration declines in diabetes compared with healthy animals (Kim et al., 2020; Nguyen et al., 2020). Furthermore, PDLSCs from aged microenvironment exhibited reduced colony-forming ability, proliferative, migratory and osteogenic potential than PDLSCs from healthy PDL (Zheng et al., 2009; Wu et al., 2015; Aung et al., 2020). For PDLSCs isolated from periodontitis, our team previously found that the proliferative capacity of them increased but the osteogenic potential of them was significantly lower than that of PDLSCs isolated from healthy PDL (Liu et al., 2011; Tang et al., 2016; Sun et al., 2017). In this study, flow cytometry analysis and colony-forming assays showed the PDLSCs we cultured from different origins expressed MSCs surface markers and owed self-renew potential, suggesting the PDLSCs we collected displayed MSCs properties (Supplementary Figures S1, S2). Then, we analyzed the osteogenic potential of PDLSCs derived from periodontitis and older donors. The findings demonstrated dysfunctional osteogenic potential, which was consistent with previous studies (Figures 1, 3A–D) (Tang et al., 2016; Sun et al., 2017).

ERM, as the descendant of HERS which are crucial in root formation, play an important role in cementum repair and periodontal homeostasis (Keinan and Cohen, 2013; Nam et al., 2014; Yang et al., 2020). During root dentine formation, HERS cells dislocate and disintegrate from the root, which allows the attachment of cementum-forming cells to the newly formed root dentin surface (Duan et al., 2020). The remaining epithelial cells are further removed from the root surface and retained in the developing periodontal membrane, which are known as ERM. Through HERS act as a transient structure, their epithelial residue ERM exist throughout life with a fish net structure surrounded the root surface (Ohshima et al., 2008). Compared with HERS, ERM cells derive from a wider variety of sources, which are ideal cells to investigate the biological function of epithelial cells in periodontal ligament (Nam et al., 2014; Athanassiou-Papaefthymiou et al., 2015). Since there are only a small amount of the ERM cells in PDL, it is difficult to obtain and culture human ERM cells. While several studies have cultured the human ERM cells *in vitro* and evaluated their characteristics (Nam et al., 2011; Lee et al., 2012; Kitajima et al., 2019). As the ERM coexist with PDL cells and close to gingival cells, the improvement of purification of the ERM is a matter of considerable interest in order to clarify the function of this subpopulation (Athanassiou-Papaefthymiou et al., 2015). Summarizing the methods of others, we mechanically scraping the periodontal tissue of upper 1/3 of root to avoid the effects of gingival epithelial cells. Additionally, enzymatic digestion of the explants culture, which contains PDL cells and ERM, allows the less-adherent periodontal ligament fibroblasts to be released and separated from the more-adherent ERM (Nam et al., 2014; Kitajima et al., 2019). However, eliminating any residual other cells from the remaining adherent ERM remains difficult which needs advanced technology such as flow cytometry sorting. Given that heterogeneity of cell types exists throughout the evolution in every functional entity (Krivanek et al., 2017), the heterogeneity in the ERM population we isolated still exists. For example, some cell tracing studies have uncovered the critical role of biomarkers such as Sox2^+^ dental epithelial stem cells in renewal of continuously growing mouse incisor (Juuri et al., 2012). Our team found that Gli1^+^ cells in PDL can sense mechanical forces and participated in periodontal remodeling (Liu et al., 2020). In the future, we will further investigate the specific biomarkers in ERM and their function. In this study, based on the experimental methods of others, we successfully obtained high purity ERM through expression of CK14^+^ cells and gene expression of epithelial markers. Our results showed that high purity ERM were obtained from human periodontal tissues which can be applied for further studies (Figure 2).

A co-culture system provides an ideal model to mimic the microenvironment *in vivo*, facilitating cell communication (Liu et al., 2014a). Several previous *in vitro* studies have demonstrated that ERM are capable of expressing extracellular matrix proteins that are involved in regulating mineralization such as osteopontin, bone sialoprotein and osteoprotegerin (Xiong et al., 2013; Yang et al., 2015). Furthermore, the osteogenic ability of PDLSCs can be enhanced by co-culture with HERS (Sonoyama et al., 2007). However, whether ERM inherit HERS function and how ERM impact PDLSCs remain largely unknown. In this study, we examined ALP and RUNX-2 as classical osteogenic markers. Although ALP and RUNX-2 are considered as early osteogenic markers, studies have showed that in the process of new bone formation, the expression of oteocalcin (OCN) which is considered as late osteogenic marker parallels with the expression of ALP. They both increase with the bone formation process and decrease with the maturation process (Diemar et al., 2021). Even so, further examination of the osteogenic markers such as OCN and osterix (OSX) are needed to confirm the influence of ERM in osteogenic differentiation of PDLSCs and the ALP activity also needs analysis. Besides, functional experiments *in vitro* and *in vivo* such as Aizarin red S staining and ectopic osteogenesis assay are also needed in future investigation.

In this study, we established a co-culture system of ERM and PDLSCs derived from different microenvironment. The data first showed that both P-PDLSCs and A-PDLSCs possessed a higher expression of ALP and RUNX-2 after co-cultured with ERM, which suggests that ERM may create a beneficial microenvironment for ameliorating the impaired osteogenic capacity of PDLSCs from an unfavorable microenvironment (Figures 3, 5). However, since the experiments were lack of the H-PDLSC control, the findings only implied that ERM could increase the impaired osteogenic capacity of PDLSCs, whether ERM improved osteogenic capacity of H-PDLSC needs further investigation.

Wnt signaling is putatively involved in various developmental processes such as skeletal development and tooth formation (Hu et al., 2019; Wang et al., 2019). It has been shown in various report that canonical Wnt signaling is activated during osteogenic differentiation in human bone marrow mesenchymal stem cells (BMMSCs) (Matsushita et al., 2020; Xiang et al., 2020). Our group has observed that a diverse regulation of the BMMSCs and PDLSCs via canonical Wnt pathway modulation (Liu et al., 2014a,b). The osteogenic differentiation process of BMMSCs activated the canonical Wnt signaling with increased level of active β-catenin and P-GSK-3β. While the osteogenic differentiation process of PDLSCs inhibited the canonical Wnt signaling with decreased level of active β-catenin and P-GSK-3β (Liu et al., 2011b; Li et al., 2016, 2018). It has been reported that the microenvironment influents the Wnt pathway (Liu et al., 2011a). For example, PDLSCs treated with the inflammatory factor TNF-α under osteogenic induction conditions exhibit the upregulation of Wnt signaling (Fang et al., 2020). Our team also detected that the application of the Wnt signaling antagonist DKK1 led to an increase in osteogenic differentiation in PDLSCs derived from the inflammatory microenvironment (Sun et al., 2017). Earlier studies have found that aging is also a chronic inflammatory process (Zhang et al., 2012; Aung et al., 2020), which implicates similar trends of cells from donors with periodontitis and aged donors. In this study, the results showed that P-GSK-3β and active-β-catenin decreased in the co-culture system, while GSK-3β and total-β-catenin remained unmodified, suggesting the suppression of the Wnt pathway in PDLSCs from an impaired microenvironment after co-cultured with ERM (Figure 6). Whereas more in-depth mechanism exploration is needed such as wheather ERM release factors to effect PDLSCs proliferation or differentiation.

In conclusion, we first found that ERM alleviated the osteogenic potential of PDLSCs from inflammatory and aged microenvironments, providing a novel function of ERM and a novel therapeutic to optimize PDLSCs derived from unfavorable microenvironments for better periodontal regenerative outcomes.

## Data Availability Statement

The raw data supporting the conclusions of this article will be made available by the authors, without undue reservation.

## Ethics Statement

The studies involving human participants were reviewed and approved by Ethics Board of School of Stomatology, the Fourth Military Medical University. The patients/participants provided their written informed consent to participate in this study.

## Author Contributions

YL and AL conceptualized and performed the experiments and wrote the manuscript. LiqZ, ZW, and NH analyzed the data. QZ and LisZ contributed to the *in vitro* experiments. ZJ and FJ conceptualized the experiments, interpreted results, and provide critical revisions of the manuscript. All authors approved the final version of the manuscript.

## Funding

This study was supported by the National Natural Science Foundation of China (Project No. 81870796).

## Conflict of Interest

The authors declare that the research was conducted in the absence of any commercial or financial relationships that could be construed as a potential conflict of interest.

## Publisher's Note

All claims expressed in this article are solely those of the authors and do not necessarily represent those of their affiliated organizations, or those of the publisher, the editors and the reviewers. Any product that may be evaluated in this article, or claim that may be made by its manufacturer, is not guaranteed or endorsed by the publisher.
