# Supplementary material for: Epithelial Cell Rests of Malassez Provide a Favorable Microenvironment for Ameliorating the Impaired Osteogenic Potential of Human Periodontal Ligament Stem Cells
Source: Front Physiol. 2021 Oct 11;12:735234. doi: 10.3389/fphys.2021.735234 (PMC8542701; doi:10.3389/fphys.2021.735234)
Supplement: Supplementary file 1 [file Data_Sheet_1.pdf]

Supplementary data

Epithelial Cell Rests of Malassez Provide a Favorable Microenvironment for Ameliorating the Impaired Osteogenic Potential of Human Periodontal Ligament Stem Cells

Yanjiao Li [1†], Anqi Liu [1,4†], Liqiang Zhang [2,3], Zhiwei Wang [1], Nana Hui [1], Qiming Zhai [1,2,3], Lishu Zhang [1,2,3], Zuolin Jin [1*], Fang Jin [1,2*]

[1]State Key Laboratory of Military Stomatology & National Clinical Research Center for Oral Diseases & Shaanxi Clinical Research Center for Oral Diseases, Department of Orthodontic， School of Stomatology, The Fourth Military Medical University, Xi'an, Shaanxi 710032, China.
[2]Center for Tissue Engineering, School of Stomatology, The Fourth Military Medical University, Xi'an, Shaanxi 710032, China.
[3]Xi'an Institute of Tissue Engineering and Regenerative Medicine, Xi'an, Shaanxi 710032, China.
[4]The 985 Hospital of PLA, Taiyuan, Shanxi 030001, China.
[†]These authors have contributed equally to this work and share first authorship.
[*]**Corresponding authors**: **Prof. Fang Jin and Prof. Zuolin Jin**
State Key Laboratory of Military Stomatology, The Fourth Military Medical University, Xi'an, Shaanxi 710032, China. *E-mails*: fangjin191@163.com (F.J.) and zuolinj@163.com (Z.J.); *Tel:* +86-029-84776472; *Fax:* +86-029-83218039.

Keywords: Periodontal ligament stromal/stem cells; Epithelial cell rests of Malassez; Osteogenesis; Coculture; Wnt pathway

MATERIALS AND METHODS

Flow Cytometry analysis

Approximately 1×10^6 PDLSCs were incubated with PE-conjugated human antibodies against CD90 and CD45 (Biolegend, 1:100), and FITC-conjugated human antibodies against CD29 and CD105 (Biolegend, 1:100). Cells were incubated in a 4 °C dark environment with these antibodies for 1 hour and observed with flow cytometer (Beckman Coulter, USA). For demarcating positive cells, nude cells were used as negative control.

Colony Forming Assays

PDLSCs single-cell suspensions ($1x10^3$ cells) were seeded in 10-cm diameter culture dishes and cultured in α-MEM supplemented with 10% FBS. After 14 days, the sample was fixed in 4% paraformaldehyde and stained with 0.1% toluidine blue.

Osteogenic Differentiation Assay

PDLSCs were cultured in α-MEM supplemented with 10% FBS. When the cells reached approximately 90% confluence, the culture medium was changed to osteoinductive medium. After 21 days of osteogenic induction, the samples were fixed for Alizarin red S staining.

FIGURE LEGENDS

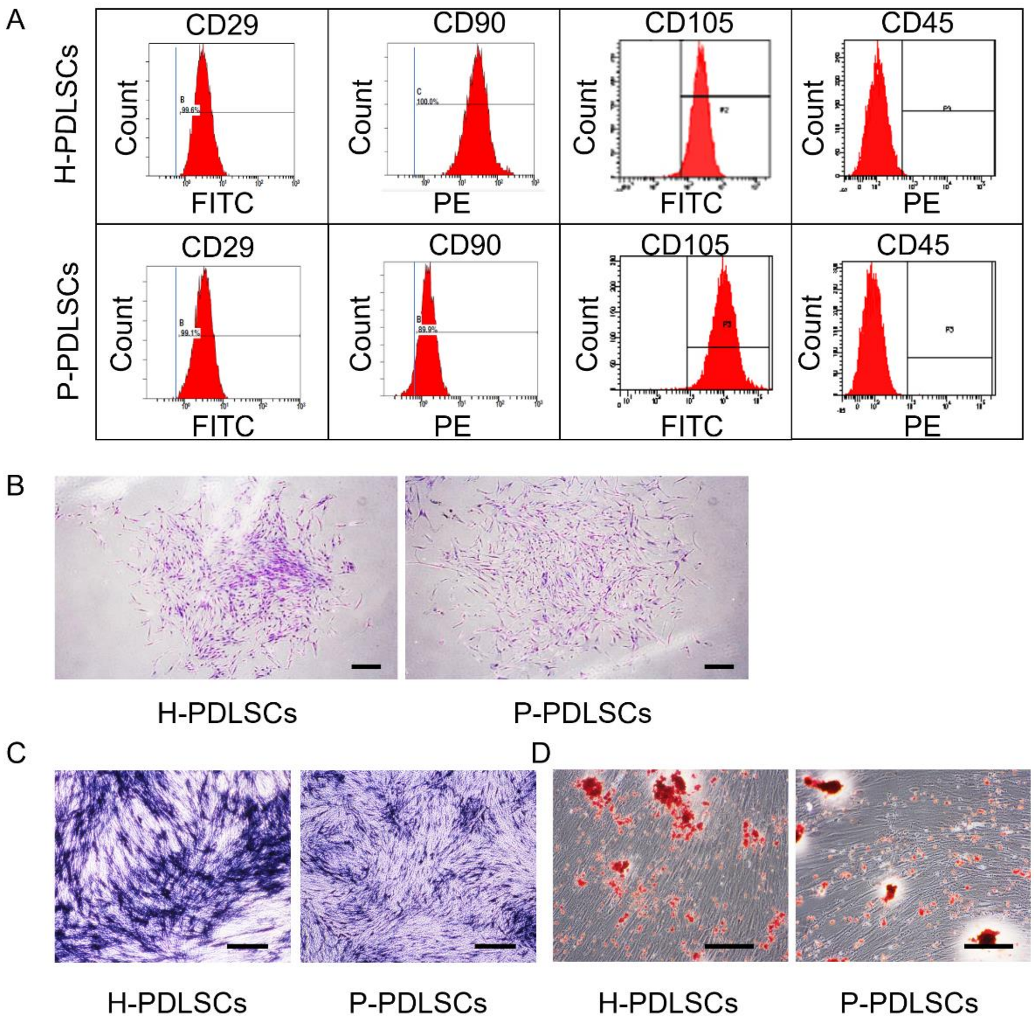

SUPPLEMENTARY FIGURE 1 Isolation and identification of H-PDLSCs and P-PDLSCs. (A) Mesenchymal stem cell phenotype examination by flow cytometric analysis. (B) Colony-formation assay of H-PDLSCs and P-PDLSCs observed by microscopy. Scale bar, 200 mm. (C) ALP staining of H-PDLSCs and P-PDLSCs observed by microscopy. Scale bar, 100 mm. (D) Alizarin red S staining shows the osteogenic nodule formation of H-PDLSCs and P-PDLSCs. Scale bar, 200 mm.

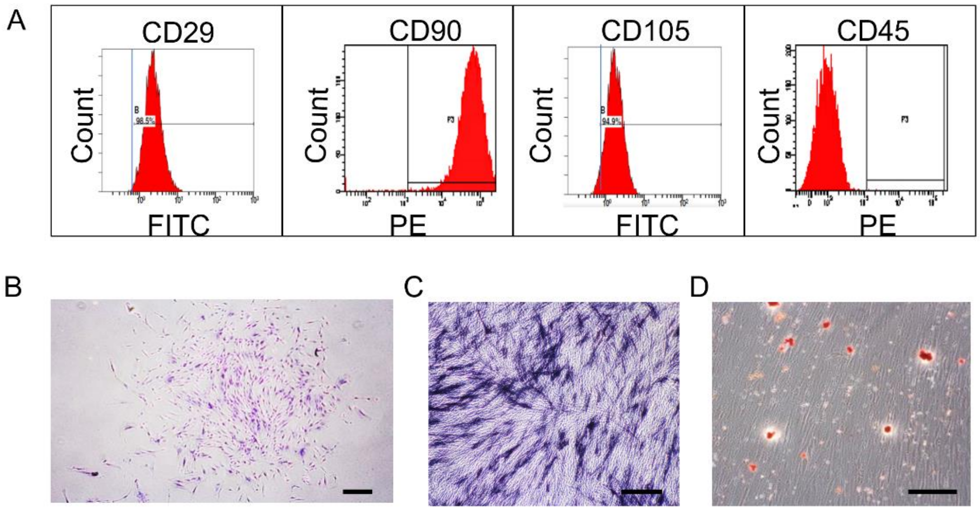

SUPPLEMENTARY FIGURE 2 Isolation and identification of A-PDLSCs. (A) Mesenchymal stem cell phenotype examination by flow cytometric analysis. (B) Colony-formation assay of A-PDLSCs observed by microscopy. Scale bar, 200 mm. (C) ALP staining of H-PDLSCs and P-PDLSCs observed by microscopy. Scale bar, 100 mm. (D) Alizarin red S staining shows the osteogenic nodule formation A-PDLSCs. Scale bar, 200 mm.
