# Supplementary figures and images for: Epithelial Cell Rests of Malassez Provide a Favorable Microenvironment for Ameliorating the Impaired Osteogenic Potential of Human Periodontal Ligament Stem Cells
Source: Front Physiol. 2021 Oct 11;12:735234. doi: 10.3389/fphys.2021.735234 (PMC8542701; doi:10.3389/fphys.2021.735234)

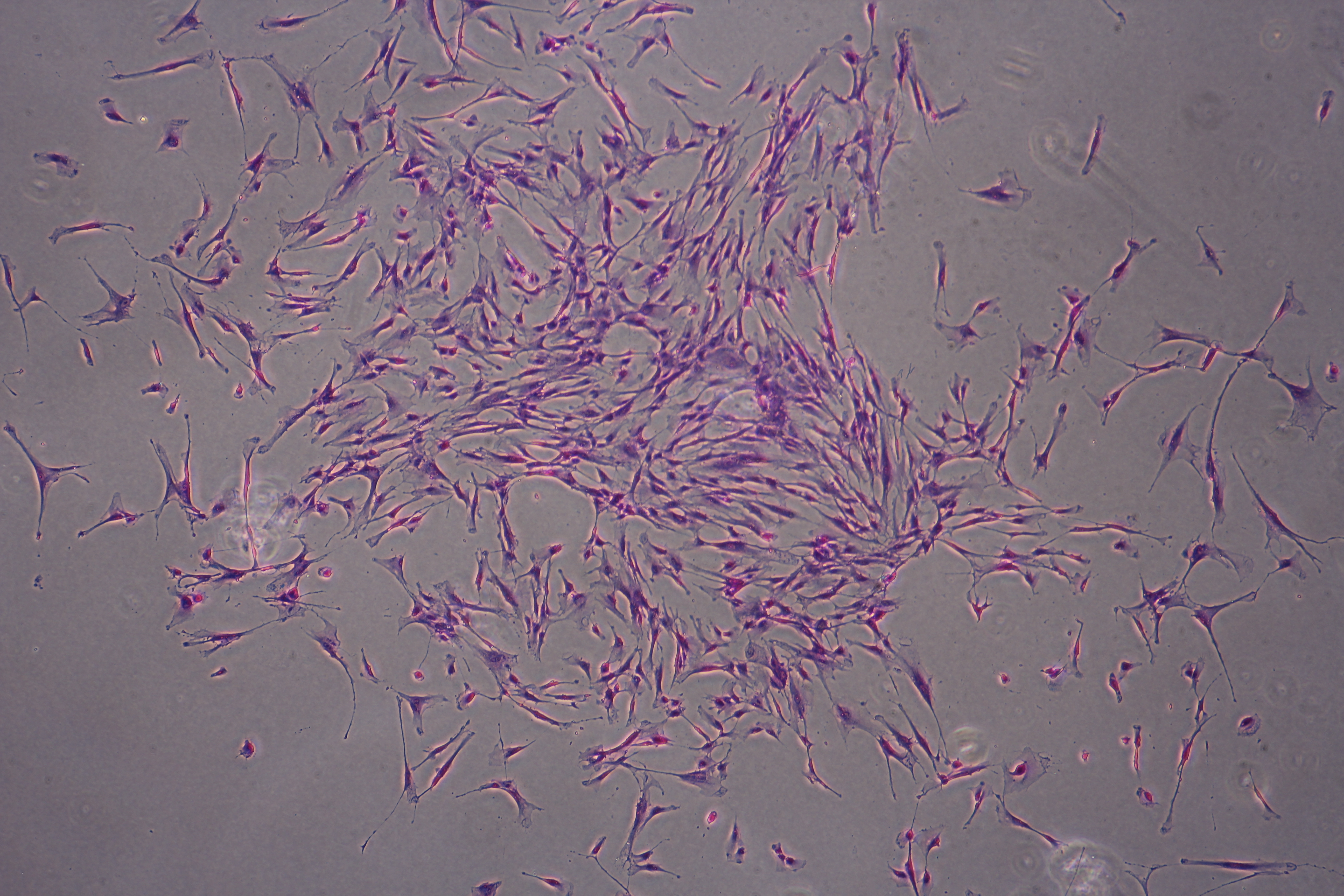

Supplement: Supplementary file 2 [file Data_Sheet_2.ZIP › Data - Supplementary/CFU/A-PDLSCs.JPG]

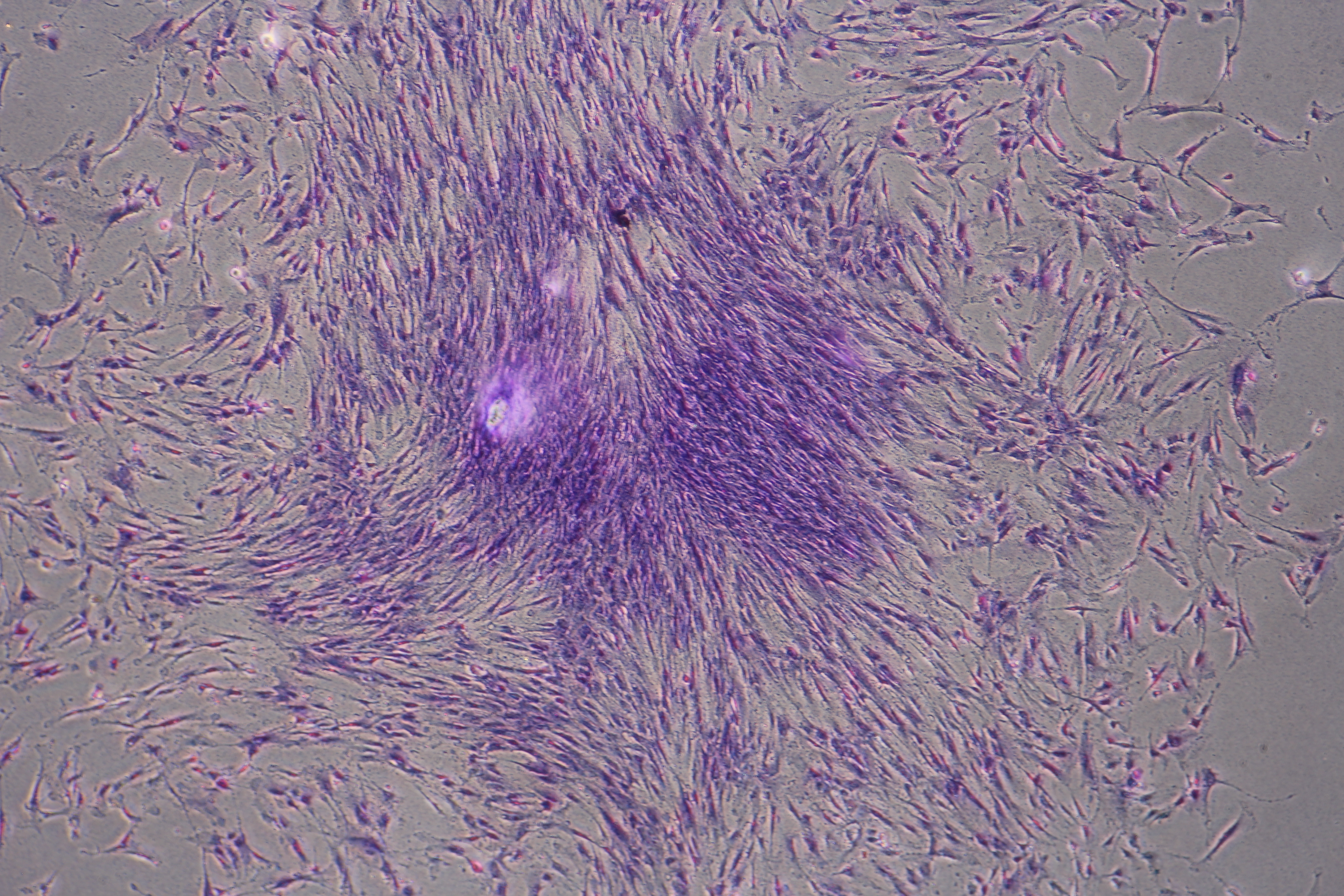

Supplement: Supplementary file 2 [file Data_Sheet_2.ZIP › Data - Supplementary/CFU/HPDLSCs.JPG]

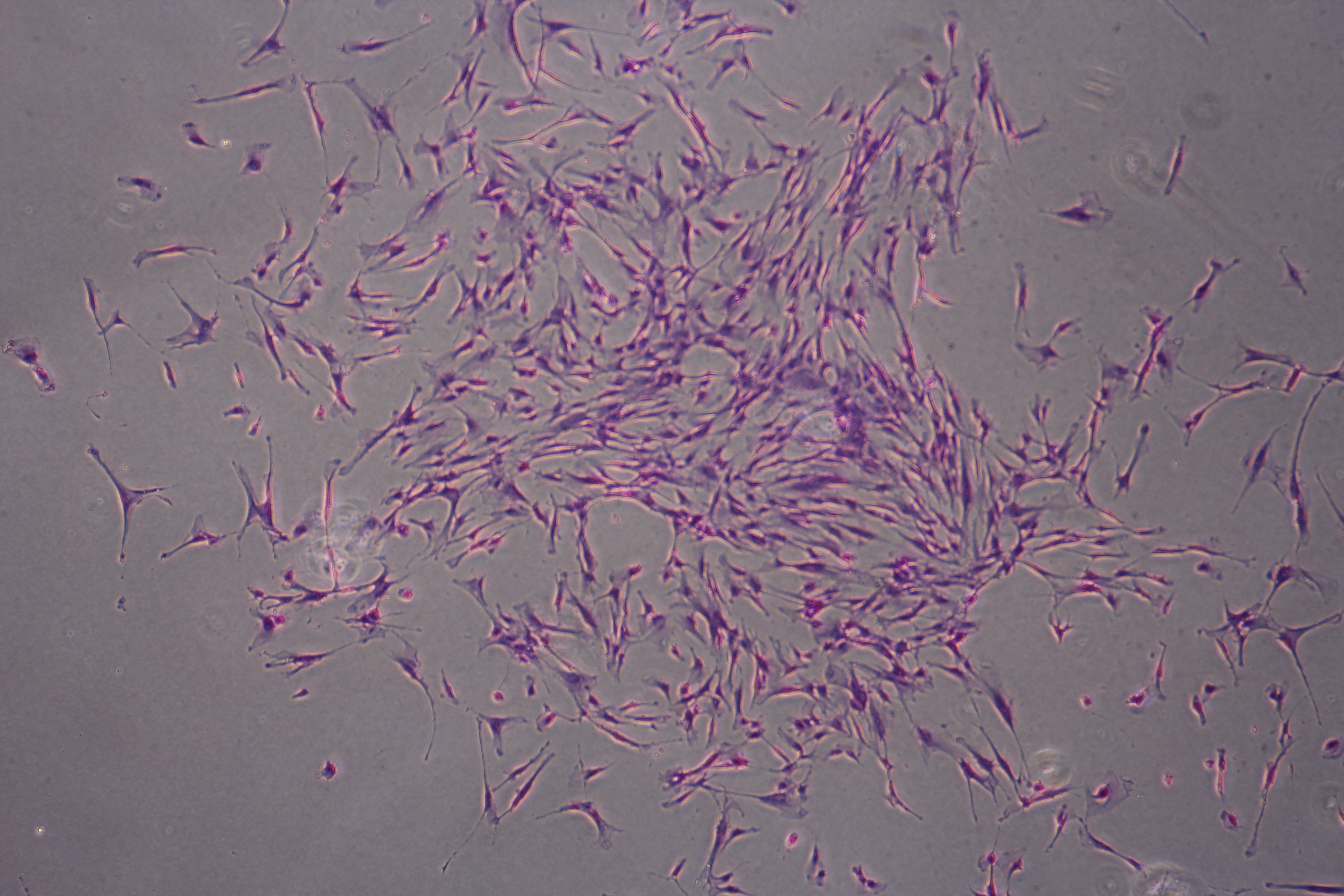

Supplement: Supplementary file 2 [file Data_Sheet_2.ZIP › Data - Supplementary/CFU/P-PDLSCs.JPG]

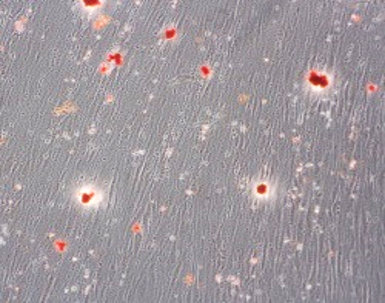

Supplement: Supplementary file 2 [file Data_Sheet_2.ZIP › Data - Supplementary/osteogenic nodule formation/A-PDLSC.tif]

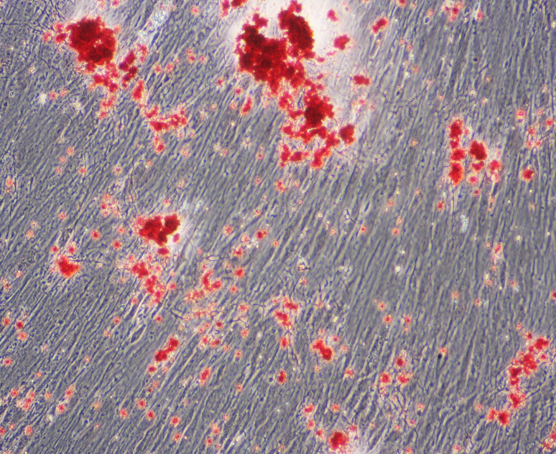

Supplement: Supplementary file 2 [file Data_Sheet_2.ZIP › Data - Supplementary/osteogenic nodule formation/H-PDLSC.tif]

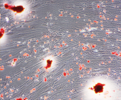

Supplement: Supplementary file 2 [file Data_Sheet_2.ZIP › Data - Supplementary/osteogenic nodule formation/P-PDLSC.tif]

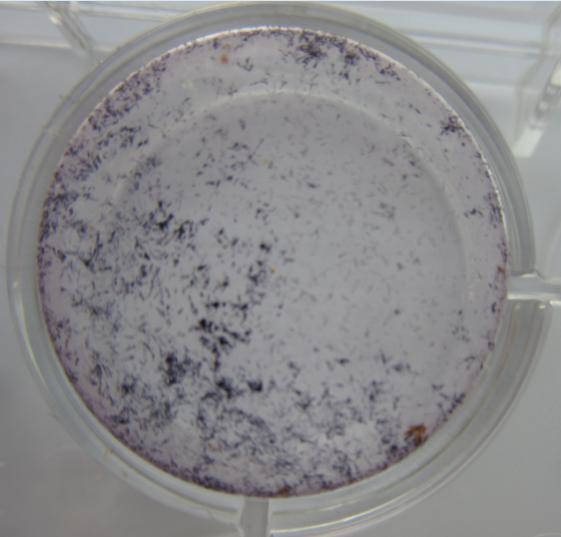

Supplement: Supplementary file 3 [file Data_Sheet_3.ZIP › Data/ALP/15-Aged-ost.jpg]

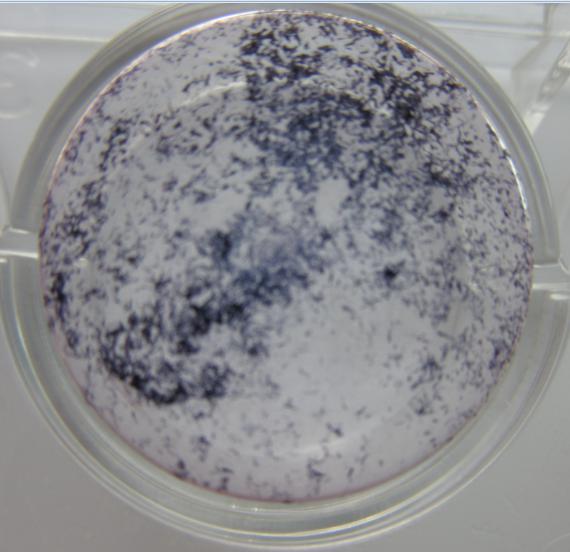

Supplement: Supplementary file 3 [file Data_Sheet_3.ZIP › Data/ALP/16-Aged-E-ost.jpg]

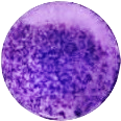

Supplement: Supplementary file 3 [file Data_Sheet_3.ZIP › Data/ALP/A-E-PDLSC.tif]

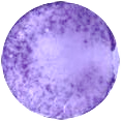

Supplement: Supplementary file 3 [file Data_Sheet_3.ZIP › Data/ALP/A-PDLSC.tif]

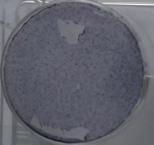

Supplement: Supplementary file 3 [file Data_Sheet_3.ZIP › Data/ALP/Aged-PDLSC.jpg]

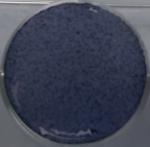

Supplement: Supplementary file 3 [file Data_Sheet_3.ZIP › Data/ALP/H-CON.jpg]

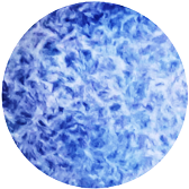

Supplement: Supplementary file 3 [file Data_Sheet_3.ZIP › Data/ALP/H-PDLSC.tif]

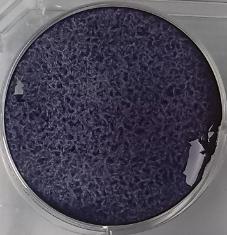

Supplement: Supplementary file 3 [file Data_Sheet_3.ZIP › Data/ALP/H-ost.jpg]

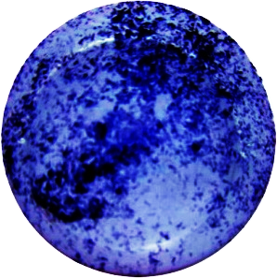

Supplement: Supplementary file 3 [file Data_Sheet_3.ZIP › Data/ALP/P-E-PDLSC-0.tif]

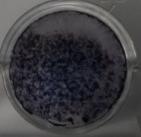

Supplement: Supplementary file 3 [file Data_Sheet_3.ZIP › Data/ALP/P-E-ost-2.jpg]

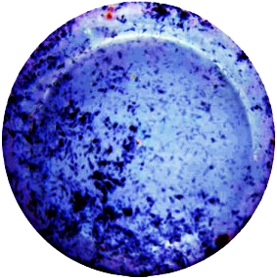

Supplement: Supplementary file 3 [file Data_Sheet_3.ZIP › Data/ALP/P-PDLSC.tif]

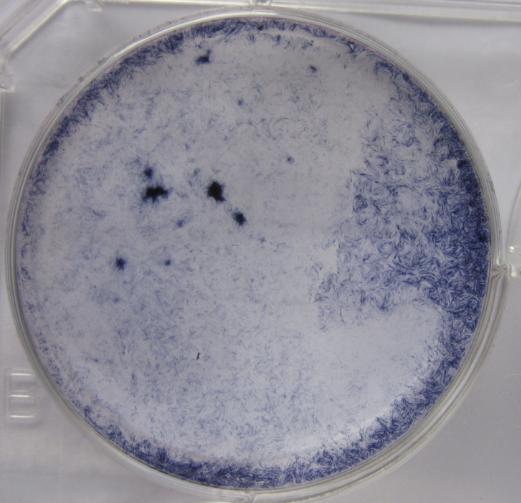

Supplement: Supplementary file 3 [file Data_Sheet_3.ZIP › Data/ALP/P-con.jpg]

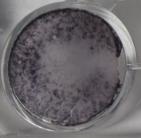

Supplement: Supplementary file 3 [file Data_Sheet_3.ZIP › Data/ALP/P-ost-2.jpg]

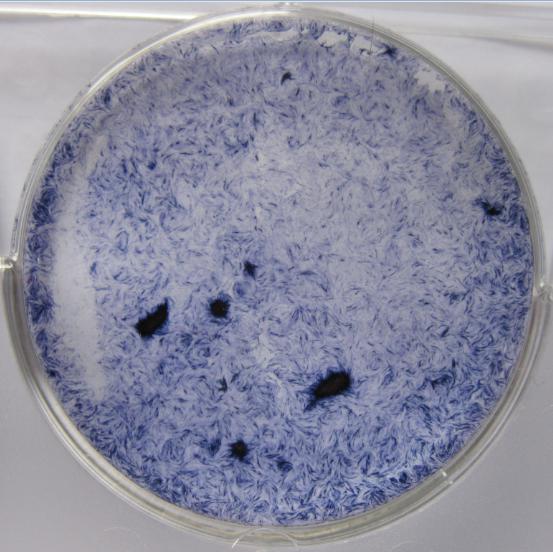

Supplement: Supplementary file 3 [file Data_Sheet_3.ZIP › Data/ALP/P-ost.jpg]

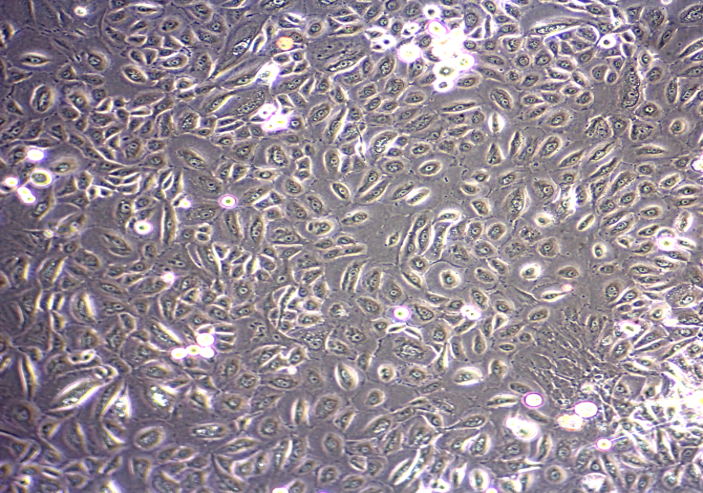

Supplement: Supplementary file 3 [file Data_Sheet_3.ZIP › Data/ERM/1.tif]

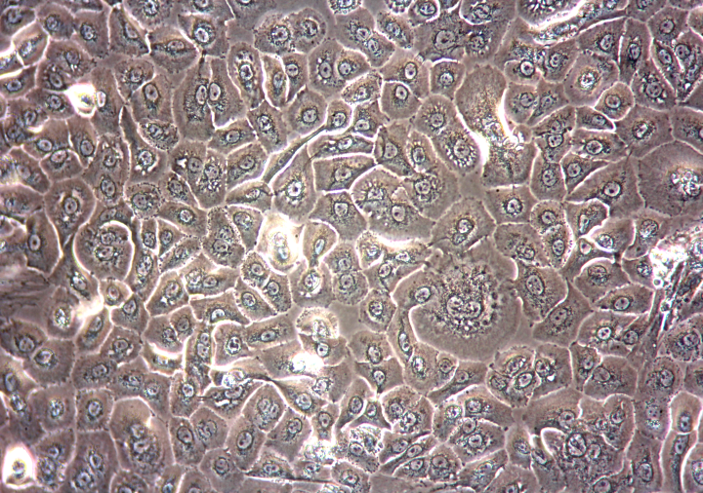

Supplement: Supplementary file 3 [file Data_Sheet_3.ZIP › Data/ERM/2.tif]

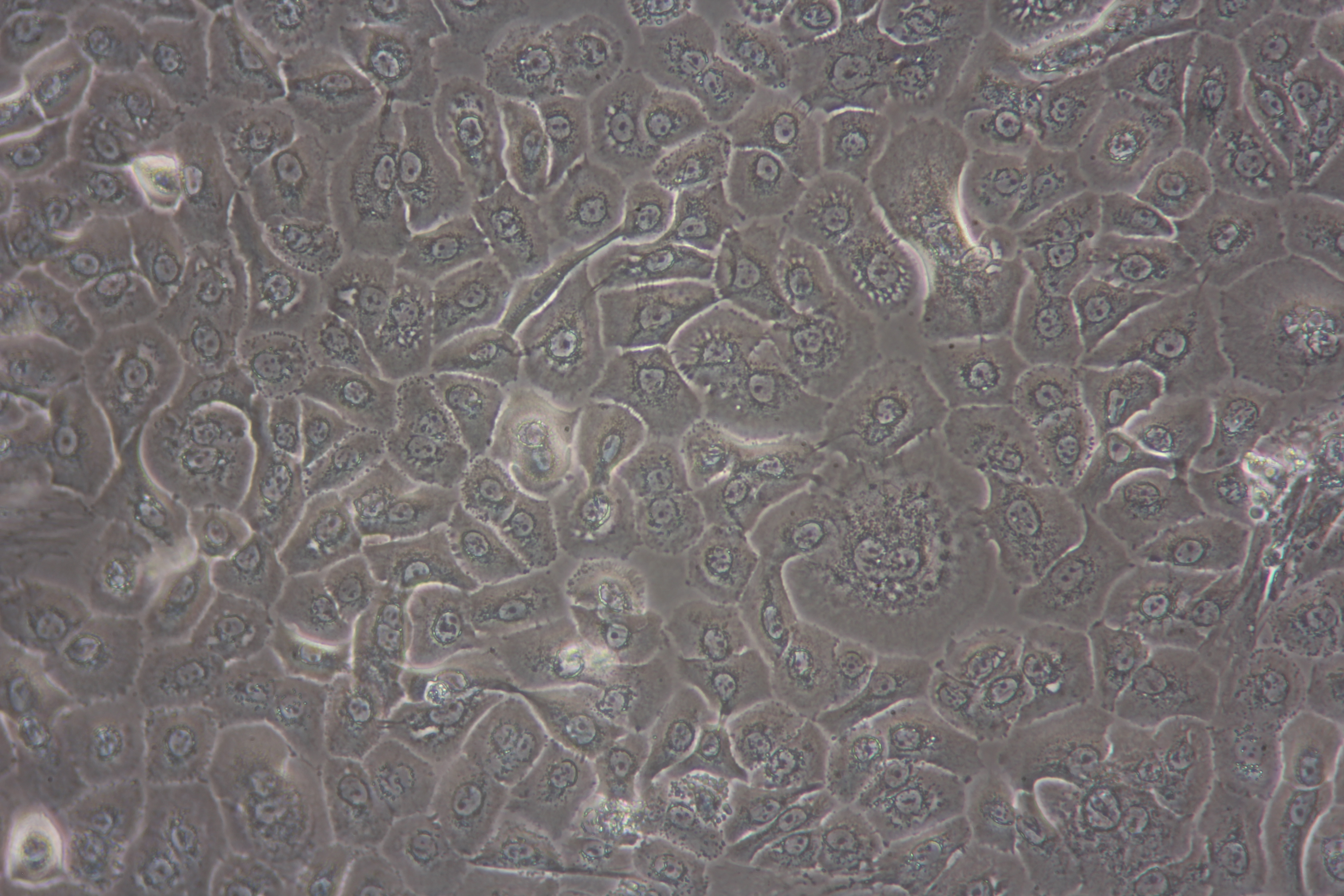

Supplement: Supplementary file 3 [file Data_Sheet_3.ZIP › Data/ERM/IMG_9043.JPG]

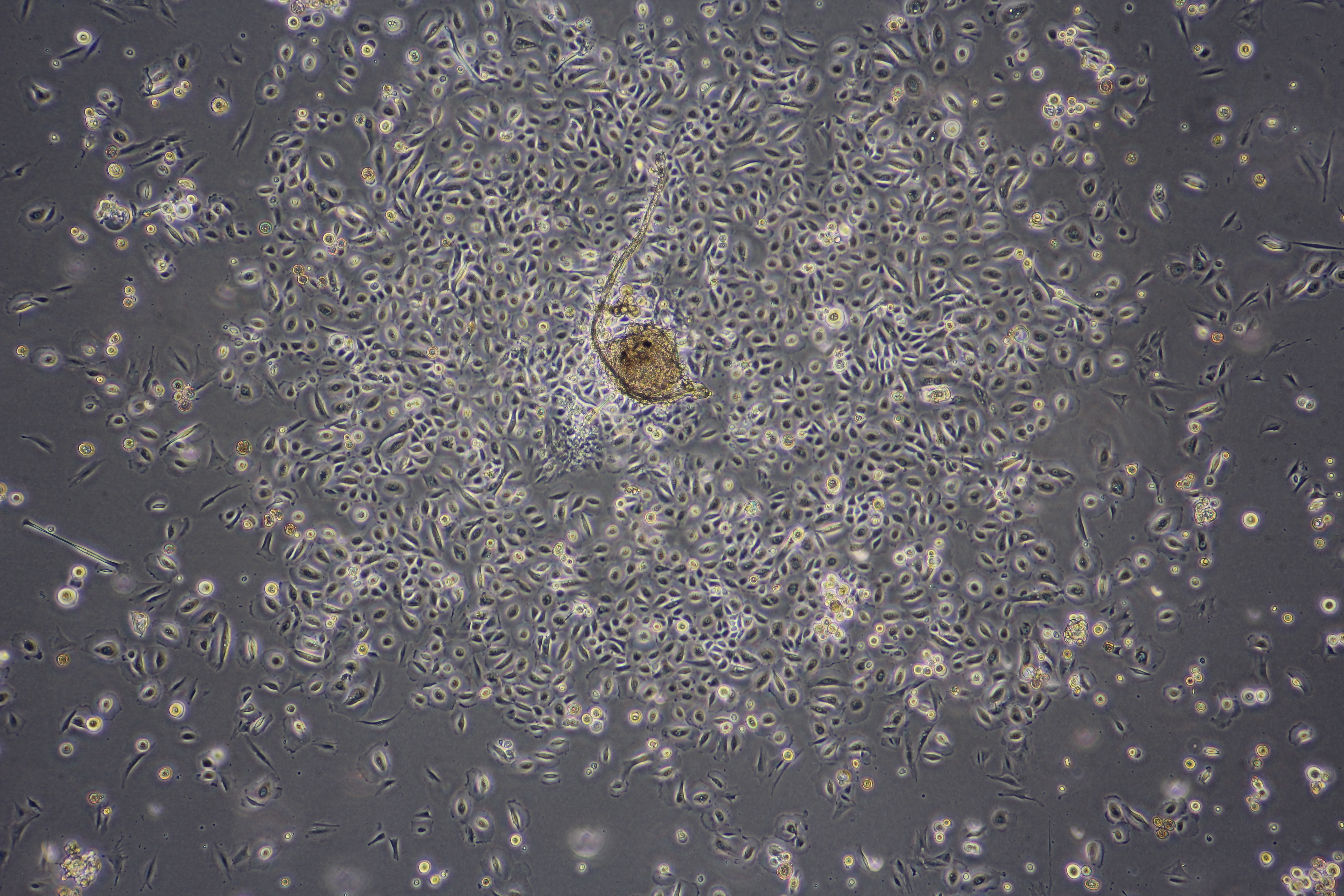

Supplement: Supplementary file 3 [file Data_Sheet_3.ZIP › Data/ERM/IMG_9500.JPG]

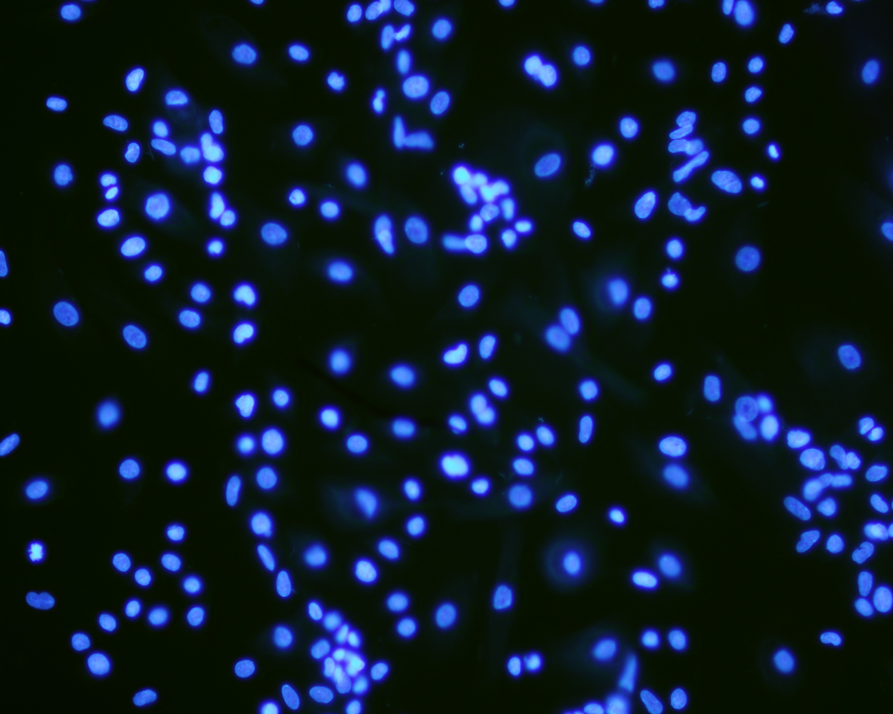

Supplement: Supplementary file 3 [file Data_Sheet_3.ZIP › Data/IF/1.tif]

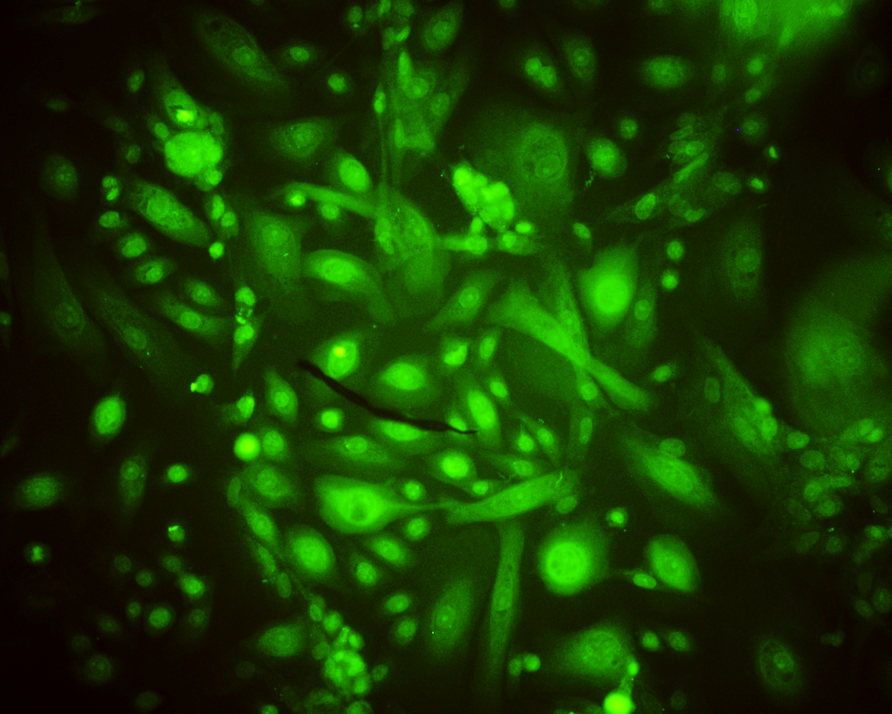

Supplement: Supplementary file 3 [file Data_Sheet_3.ZIP › Data/IF/2.tif]

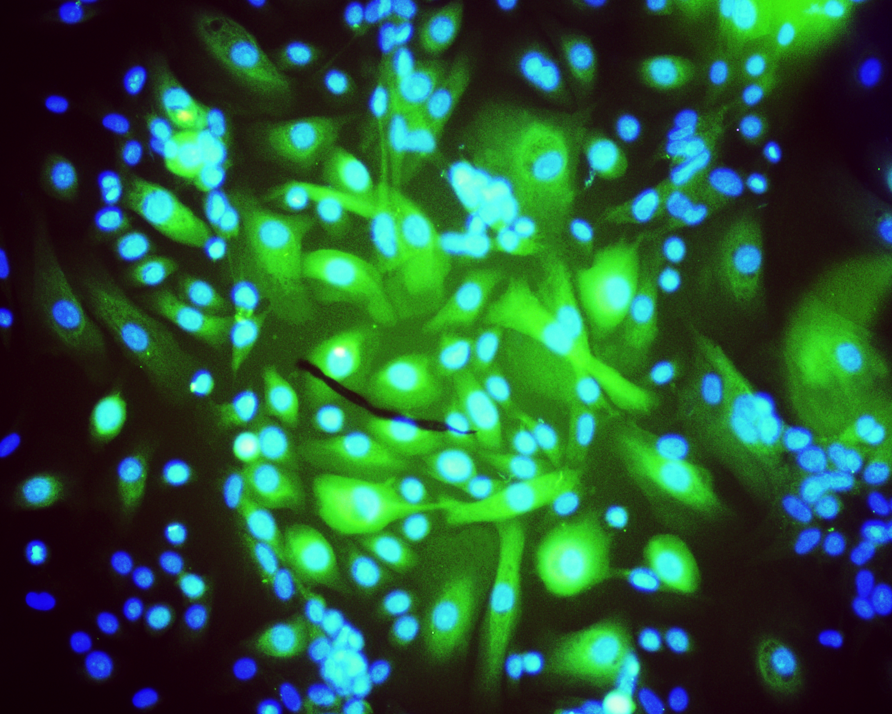

Supplement: Supplementary file 3 [file Data_Sheet_3.ZIP › Data/IF/3.tif]

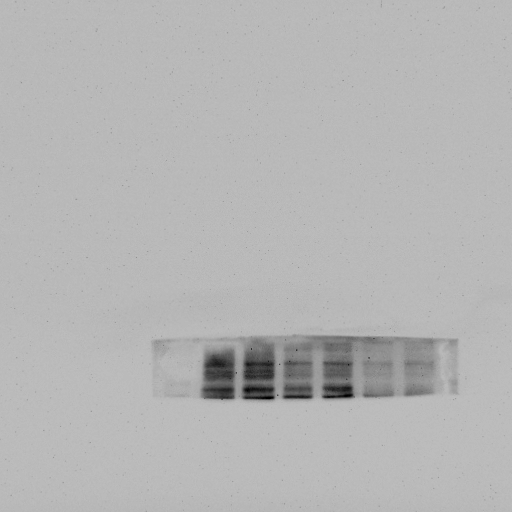

Supplement: Supplementary file 3 [file Data_Sheet_3.ZIP › Data/W.B data/ALP(56KDa)/ALP(1) (H-conú1⁄4H-ostú1⁄4P-conú1⁄4P-ost,A-PDLSCs,H-PDLSCs).jpg]

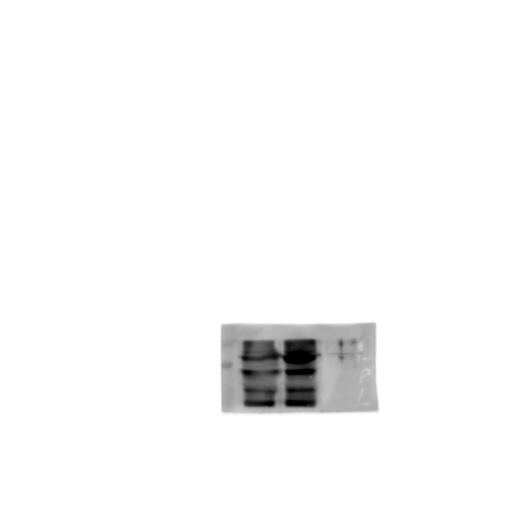

Supplement: Supplementary file 3 [file Data_Sheet_3.ZIP › Data/W.B data/ALP(56KDa)/ALP(1)(A-PDLSCs,A-PDLSCs+ERM).jpg]

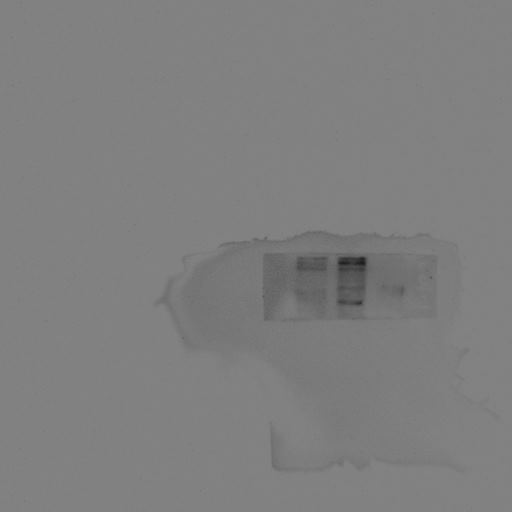

Supplement: Supplementary file 3 [file Data_Sheet_3.ZIP › Data/W.B data/ALP(56KDa)/ALP(1)(A-PDLSCs,H-PDLSCs).jpg]

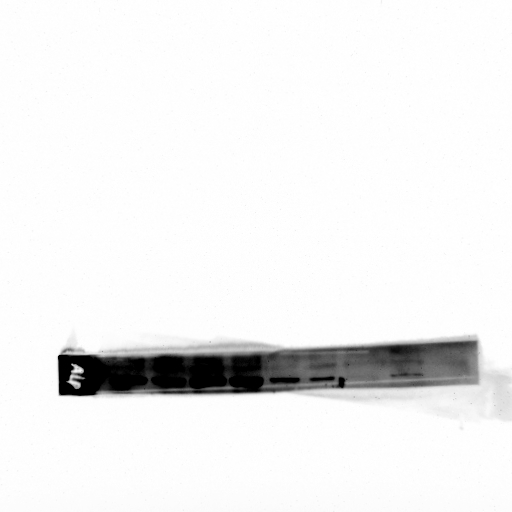

Supplement: Supplementary file 3 [file Data_Sheet_3.ZIP › Data/W.B data/ALP(56KDa)/ALP(1)(H-con,H-ost,P-con,P-ost;P-PDLSCs,P-PDLSCs+ERM).jpg]

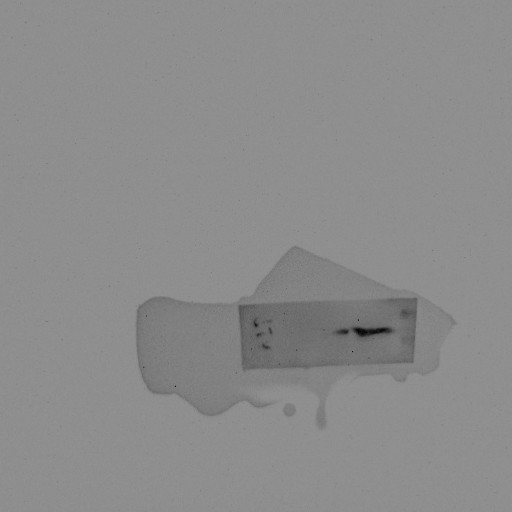

Supplement: Supplementary file 3 [file Data_Sheet_3.ZIP › Data/W.B data/ALP(56KDa)/ALP(2)(A-PDLSCs,A-PDLSCs+ERM).jpg]

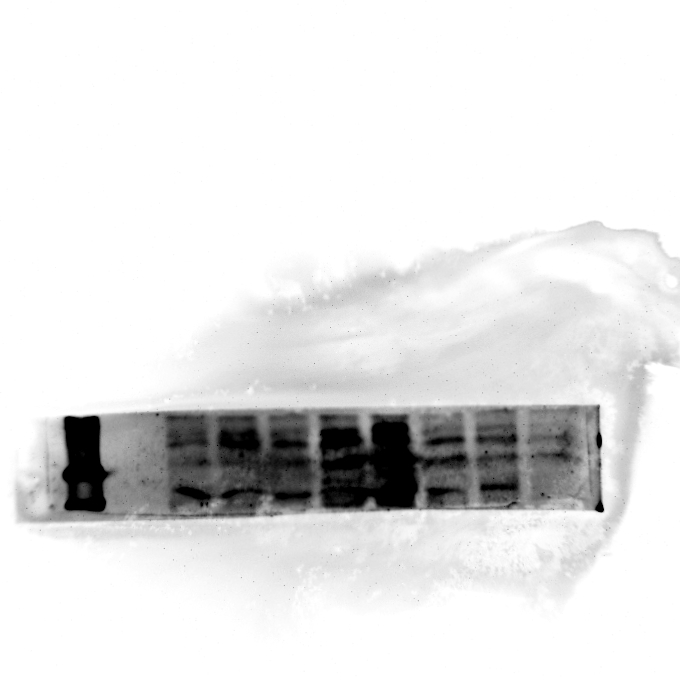

Supplement: Supplementary file 3 [file Data_Sheet_3.ZIP › Data/W.B data/ALP(56KDa)/ALP(2)(H-PDLSCs,A-PDLSCs,A-PDLSCs+ERM,H-con,H-ost,P-con,P-ost).jpg]

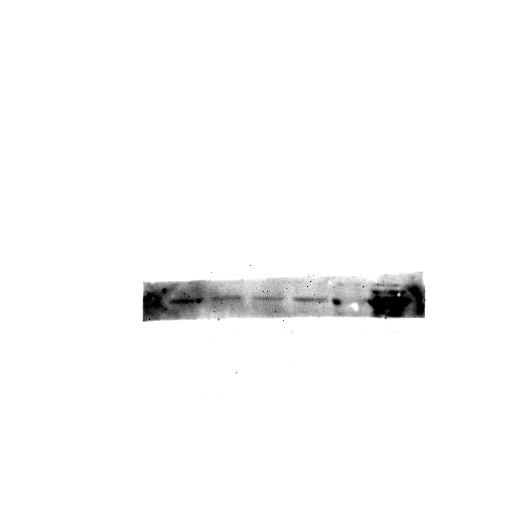

Supplement: Supplementary file 3 [file Data_Sheet_3.ZIP › Data/W.B data/ALP(56KDa)/ALP(2)(H-con,H-ost,P-con,P-ost;P-PDLSCs,P-PDLSCs+ERM).tif]

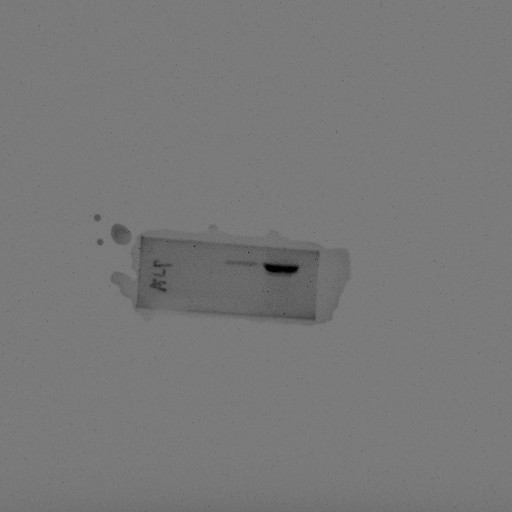

Supplement: Supplementary file 3 [file Data_Sheet_3.ZIP › Data/W.B data/ALP(56KDa)/ALP(3)(P-PDLSCs,P-PDLSCs+ERM).jpg]

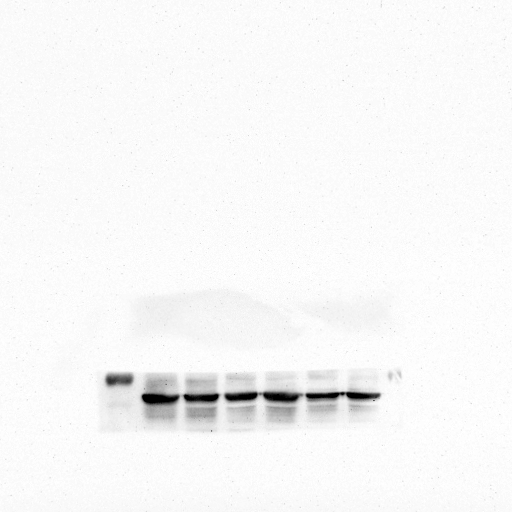

Supplement: Supplementary file 3 [file Data_Sheet_3.ZIP › Data/W.B data/ALP(56KDa)/ALP(4)ú¿H-PDLSCs,A-PDLSCs;H-conú1⁄4H-ostú1⁄4P-conú1⁄4P-ostú⌐.jpg]

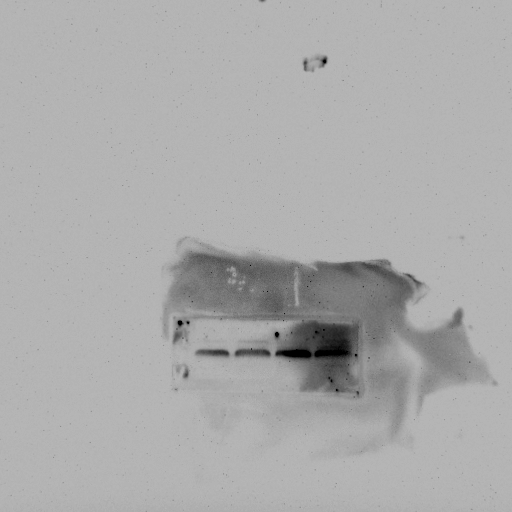

Supplement: Supplementary file 3 [file Data_Sheet_3.ZIP › Data/W.B data/GSK-3a┬ (46KDa)/GSK-3a┬(1)(P-PDLSCs,P-PDLSCs+ERM,A-PDLSCs,A-PDLSCs+ERM).jpg]

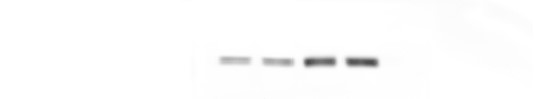

Supplement: Supplementary file 3 [file Data_Sheet_3.ZIP › Data/W.B data/GSK-3a┬ (46KDa)/GSK-3a┬(2)(P-PDLSCs,P-PDLSCs+ERM,A-PDLSCs,A-PDLSCs+ERM).jpg]

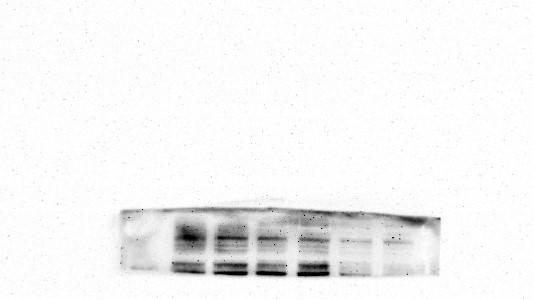

Supplement: Supplementary file 3 [file Data_Sheet_3.ZIP › Data/W.B data/GSK-3a┬ (46KDa)/GSK-3a┬(3)(P-PDLSCs,P-PDLSCs+ERM,A-PDLSCs,A-PDLSCs+ERM).jpg]

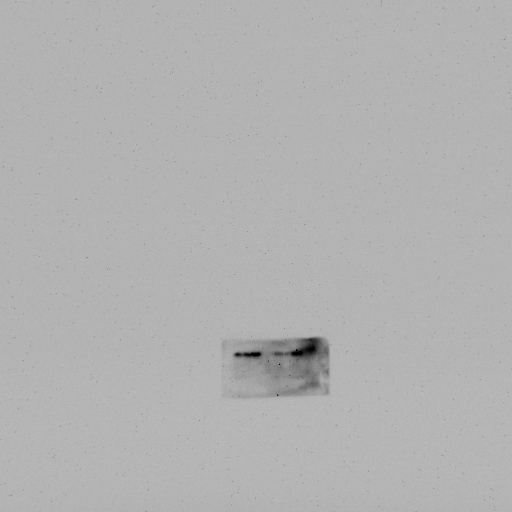

Supplement: Supplementary file 3 [file Data_Sheet_3.ZIP › Data/W.B data/P-GSK-3a┬ (46KDa)/P-GSK-3a┬(1)(A-PDLSCs,A-PDLSCs+ERM).jpg]

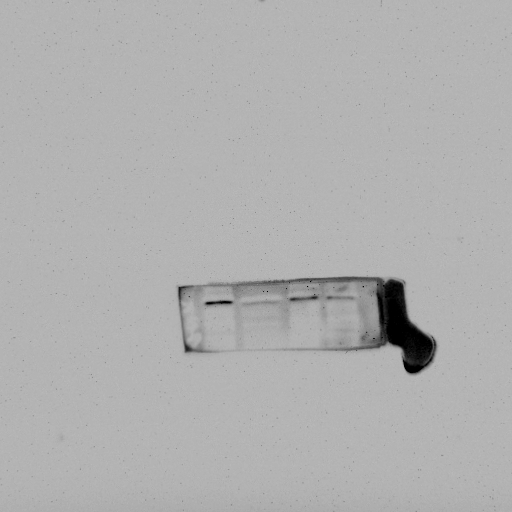

Supplement: Supplementary file 3 [file Data_Sheet_3.ZIP › Data/W.B data/P-GSK-3a┬ (46KDa)/P-GSK-3a┬(1)(A-PDLSCs,A-PDLSCs+ERM,P-PDLSCs,P-PDLSCs+ERM).jpg]

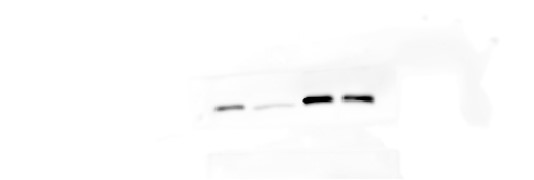

Supplement: Supplementary file 3 [file Data_Sheet_3.ZIP › Data/W.B data/P-GSK-3a┬ (46KDa)/P-GSK-3a┬(2)(A-PDLSCs,A-PDLSCs+ERM,P-PDLSCs,P-PDLSCs+ERM).jpg]

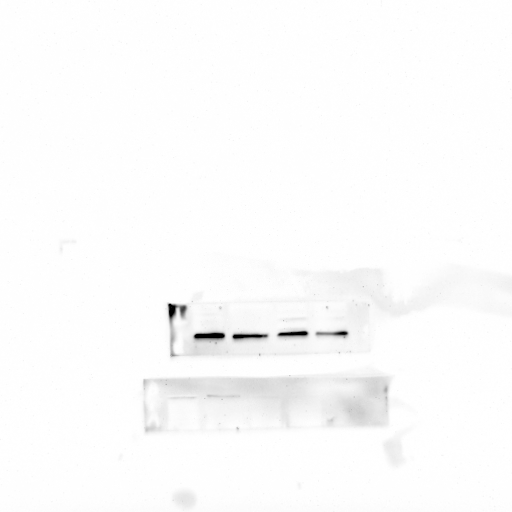

Supplement: Supplementary file 3 [file Data_Sheet_3.ZIP › Data/W.B data/P-GSK-3a┬ (46KDa)/P-GSK-3a┬(3)(A-PDLSCs,A-PDLSCs+ERM).jpg]

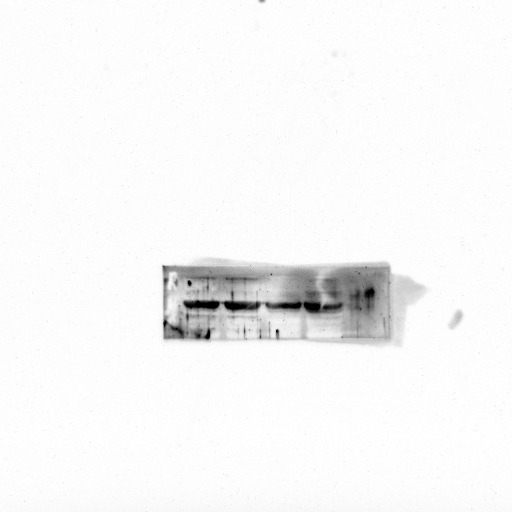

Supplement: Supplementary file 3 [file Data_Sheet_3.ZIP › Data/W.B data/Runx-2(57KDa)/Runx-2(1) (H-conú1⁄4H-ostú1⁄4P-conú1⁄4P-ost).jpg]

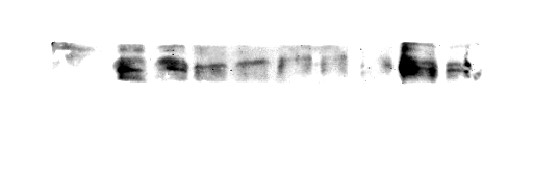

Supplement: Supplementary file 3 [file Data_Sheet_3.ZIP › Data/W.B data/Runx-2(57KDa)/Runx-2(1)(A-PDLSCs,A-PDLSCs+ERM,P-PDLSCs,P-PDLSCs+ERM,H-PDLSCs,A-PDLSCs).jpg]

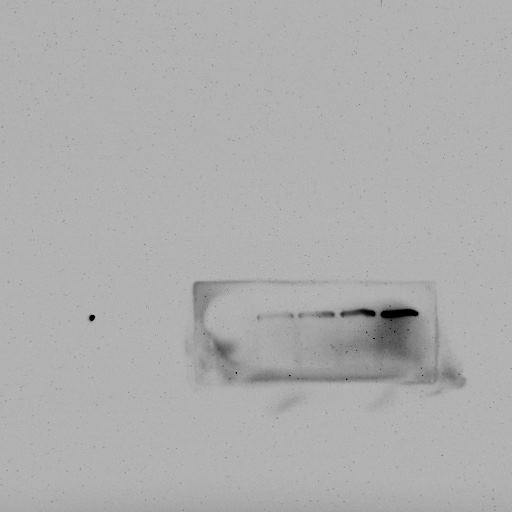

Supplement: Supplementary file 3 [file Data_Sheet_3.ZIP › Data/W.B data/Runx-2(57KDa)/Runx-2(1)(A-PDLSCs,A-PDLSCs,P-PDLSCs,P-PDLSCs+ERM).jpg]

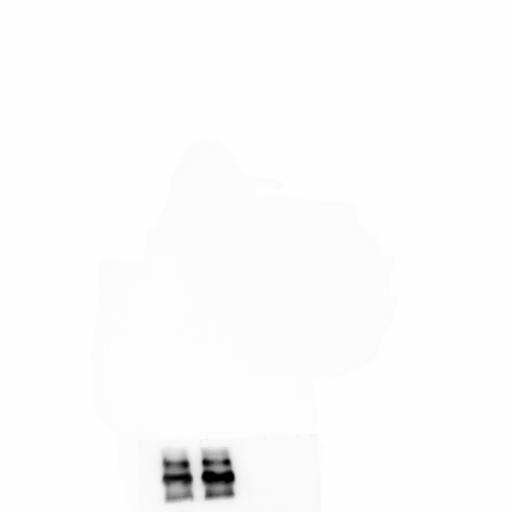

Supplement: Supplementary file 3 [file Data_Sheet_3.ZIP › Data/W.B data/Runx-2(57KDa)/Runx-2(2)(A-PDLSCs,A-PDLSCs+ERM).jpg]

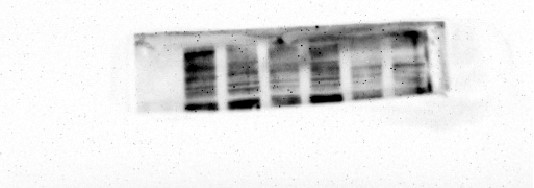

Supplement: Supplementary file 3 [file Data_Sheet_3.ZIP › Data/W.B data/Runx-2(57KDa)/Runx-2(2)(H-con,H-ost,P-con,P-ost;A-PDLSCs,H-PDLSCs).jpg]

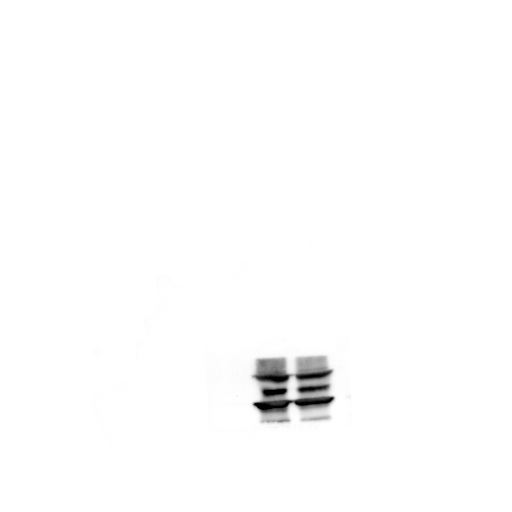

Supplement: Supplementary file 3 [file Data_Sheet_3.ZIP › Data/W.B data/Runx-2(57KDa)/Runx-2(3)(H-PDLSCs,A-PDLSCs).jpg]

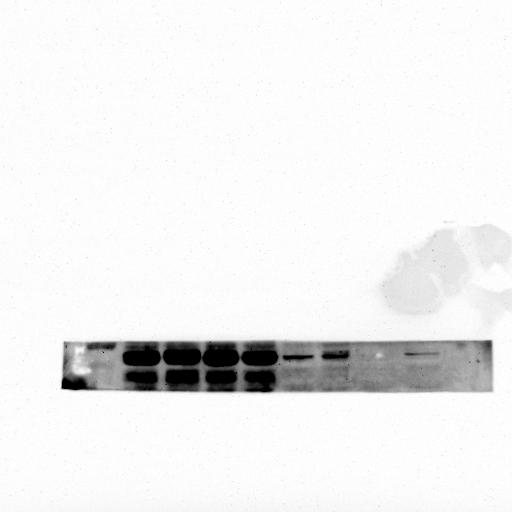

Supplement: Supplementary file 3 [file Data_Sheet_3.ZIP › Data/W.B data/Runx-2(57KDa)/Runx-2(3)(H-con,H-ost,P-con,P-ost;P-PDLSCs,P-PDLSCs+ERM).jpg]

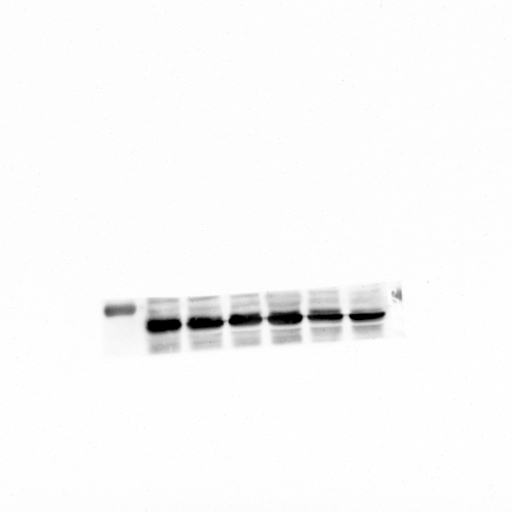

Supplement: Supplementary file 3 [file Data_Sheet_3.ZIP › Data/W.B data/Runx-2(57KDa)/Runx-2(4)(H-PDLSCs,A-PDLSCs;H-conú1⁄4H-ostú1⁄4P-conú1⁄4P-ostú⌐.jpg]

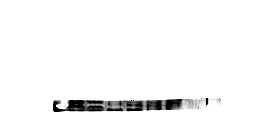

Supplement: Supplementary file 3 [file Data_Sheet_3.ZIP › Data/W.B data/Runx-2(57KDa)/Runx-2(4)(P-PDLSCs,P-PDLSCs+ERM,A-PDLSCs,A-PDLSCs+ERM,A-PDLSCs,H-PDLSCs).jpg]

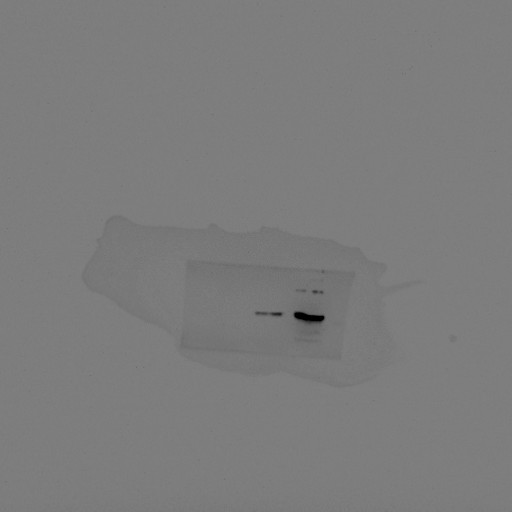

Supplement: Supplementary file 3 [file Data_Sheet_3.ZIP › Data/W.B data/a┬-catenin(active) (92KDa)/a┬-catenin(active)(1)(A-PDLSCs+ERM,A-PDLSCs).jpg]

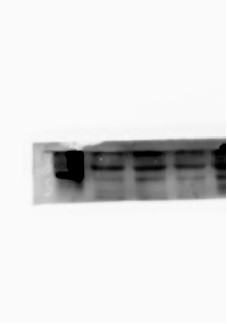

Supplement: Supplementary file 3 [file Data_Sheet_3.ZIP › Data/W.B data/a┬-catenin(active) (92KDa)/a┬-catenin(active)(1)(P-PDLSCs,P-PDLSCs+ERM,A-PDLSCs,A-PDLSCs+ERM).jpg]

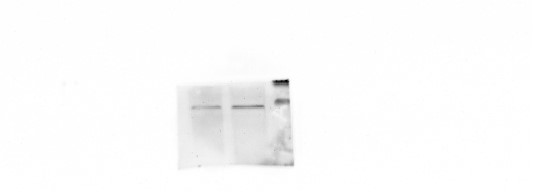

Supplement: Supplementary file 3 [file Data_Sheet_3.ZIP › Data/W.B data/a┬-catenin(active) (92KDa)/a┬-catenin(active)(2)(A-PDLSCs+ERM,A-PDLSCs).jpg]

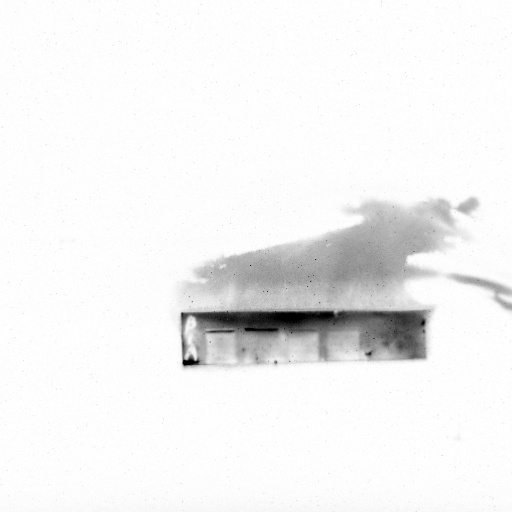

Supplement: Supplementary file 3 [file Data_Sheet_3.ZIP › Data/W.B data/a┬-catenin(active) (92KDa)/a┬-catenin(active)(2)(P-PDLSCs,P-PDLSCs+ERM,A-PDLSCs,A-PDLSCs+ERM).jpg]

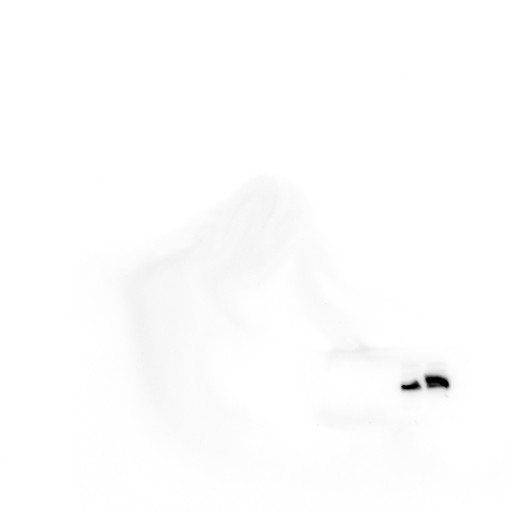

Supplement: Supplementary file 3 [file Data_Sheet_3.ZIP › Data/W.B data/a┬-catenin(active) (92KDa)/a┬-catenin(active)(3)(P-PDLSCs,P-PDLSCs+ERM,A-PDLSCs,A-PDLSCs+ERM).jpg]

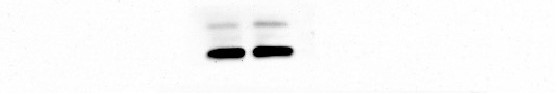

Supplement: Supplementary file 3 [file Data_Sheet_3.ZIP › Data/W.B data/a┬-catenin(total) (92KDa)/a┬-catenin(total)(1)(A-PDLSCs,A-PDLSCs+ERM).jpg]

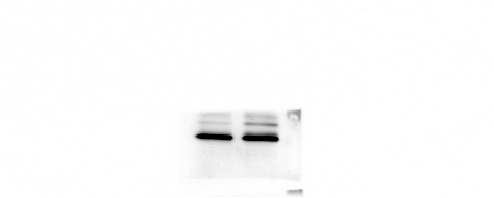

Supplement: Supplementary file 3 [file Data_Sheet_3.ZIP › Data/W.B data/a┬-catenin(total) (92KDa)/a┬-catenin(total)(1)(P-PDLSCs,P-PDLSCs+ERM).jpg]

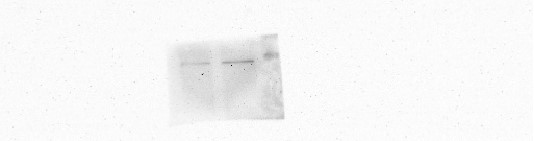

Supplement: Supplementary file 3 [file Data_Sheet_3.ZIP › Data/W.B data/a┬-catenin(total) (92KDa)/a┬-catenin(total)(2)(A-PDLSCs,A-PDLSCs+ERM).jpg]

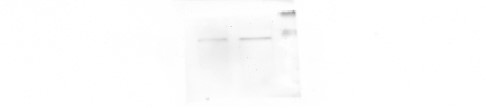

Supplement: Supplementary file 3 [file Data_Sheet_3.ZIP › Data/W.B data/a┬-catenin(total) (92KDa)/a┬-catenin(total)(2)(P-PDLSCs,P-PDLSCs+ERM).jpg]

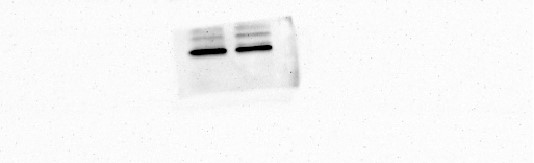

Supplement: Supplementary file 3 [file Data_Sheet_3.ZIP › Data/W.B data/a┬-catenin(total) (92KDa)/a┬-catenin(total)(3)(A-PDLSCs,A-PDLSCs+ERM).jpg]

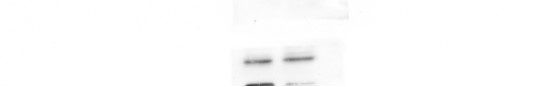

Supplement: Supplementary file 3 [file Data_Sheet_3.ZIP › Data/W.B data/a┬-catenin(total) (92KDa)/a┬-catenin(total)(3)(P-PDLSCs,P-PDLSCs+ERM).jpg]

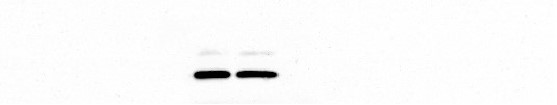

Supplement: Supplementary file 3 [file Data_Sheet_3.ZIP › Data/W.B data/a┬-catenin(total) (92KDa)/a┬-catenin(total)(4)(P-PDLSCs,P-PDLSCs+ERM).jpg]
